# Uncovering the Anti-Angiogenic Mechanisms of *Centella asiatica* via Network Pharmacology and Experimental Validation

**DOI:** 10.3390/molecules29020362

**Published:** 2024-01-11

**Authors:** Bingtian Zhao, Yuanyuan Li, Binya Wang, Jing Liu, Yang Yang, Qianghua Quan, Quan An, Rong Liang, Chunhuan Liu, Cheng Yang

**Affiliations:** 1 Key Laboratory of Synthetic and Biological Colloids, Ministry of Education, School of Chemical and Material Engineering, Jiangnan University, Wuxi 214122, China; liyuanyuan0918@163.com (Y.L.); dxsxzyzs@163.com (B.W.); lj15735649029@163.com (J.L.); rongliang@jiangnan.edu.cn (R.L.); liuch@jiangnan.edu.cn (C.L.); 2 Yunnan Baiyao Group Shanghai Science & Technology Co., Ltd., Shanghai 201100, China; doubleyoung75@163.com (Y.Y.); 18811781508@163.com (Q.Q.); ynbyanquan@sina.com (Q.A.); 3 East Asia Skin Health Research Center, Beijing 100037, China

**Keywords:** *Centella asiatica*, anti-angiogenesis, cancer, network pharmacology, triterpene

## Abstract

Background: *Centella asiatica* (CA) has been used to address cancer for centuries in traditional Chinese medicine (TCM). Previous studies demonstrated its anti-angiogenesis efficacy, but the underlying mechanism of its action remains to be further clarified. This study aims to investigate the underlying mechanisms of CA and its triterpenes in anti-angiogenesis for cancer therapeutics through network pharmacology and experimental validation. Methods: Cytoscape was used to construct a network of compound–disease targets and protein–protein interactions (PPIs) from which core targets were identified. GO and KEGG analyses were performed using Metascape, and the AutoDock-Vina program was used to realize molecular docking for further verification. Then, VEGF165 was employed to establish an induced angiogenesis model. The anti-angiogenic effects of CA were evaluated through assays measuring cell proliferation, migration, and tubular structure formation. Results: Twenty-five active ingredients in CA had potential targets for anti-angiogenesis including madecassoside, asiaticoside, madecassic acid, asiatic acid, and asiaticoside B. In total, 138 potential targets for CA were identified, with 19 core targets, including STAT3, SRC, MAPK1, and AKT1. A KEGG analysis showed that CA is implicated in cancer-related pathways, specifically PD-1 and AGE-RAGE. Molecular docking verified that the active components of CA have good binding energy with the first four important targets of angiogenesis. In experimental validation, the extracts and triterpenes of CA improved VEGF165-induced angiogenesis by reducing the proliferation, migration, and tube formation of human umbilical vein endothelial cells (HUVECs). Conclusions: Our results initially demonstrate the effective components and great anti-angiogenic activity of CA. Evidence of the satisfactory anti-angiogenic action of the extracts and triterpenes from CA was verified, suggesting CA’s significant potential as a prospective agent for the therapy of cancer.

## 1. Introduction

Angiogenesis is a hallmark of tumor development and metastasis, enabling the transportation of oxygen and nutrients to excessively proliferating tumor cells. Simultaneously, the emergence of new vessels provides a route for tumor cells to enter the bloodstream, leading to hematogenous metastasis [1]. At present, many anti-angiogenic targeted drugs are used in the treatment of malignant tumors, such as the vascular endothelial growth factor (VEGF) monoclonal antibody-Bevacizumab, oligonucleotide aptamers-Pegaptanib, recombinant fusion proteins-Aflibercept, and tyrosine kinase inhibitors. Unfortunately, most patients have many toxic reactions to these drugs and are prone to drug resistance [2]. In addition, there are some other problems, such as varied curative effects, high prices, and so on [3]. Many clinical studies have confirmed that TCM has fewer side effects and better therapeutic effects on anti-angiogenesis and inhibition of tumor metastasis through multi-link and multi-target process [4]. Therefore, the application of safe and effective TCM in cancer treatment needs to be further explored.

*Centella asiatica* (CA) is an umbelliferous plant used as medicine with the whole herb, which was first mentioned in the *Shennong Classic of Materia Medica*. It comprises various pentacyclic triterpenoids, including asiaticoside, madecassoside, madecassic acid, and asiatic acid. Additionally, it contains flavonoids such as quercetin and kaempferol [5]. CA and its extracts have the function of dispelling heat and resolving dampness, have detoxification and anti-inflammation properties, and are also recommended for the therapy of some skin conditions such as wounds, ulcers, and eczema [6]. A previous study confirmed that asiatic acid could inhibit the metastatic properties of renal cancer cells and p-ERK/p-p38MAPK expression [7]. Asiatic acid was also demonstrated to have a cancer-related cytotoxic effect which works in a dose- and time-dependent manner on cisplatin-resistant nasopharyngeal carcinoma cells [8]. Madecassic acid could alleviate colitis-associated colorectal cancer by blocking the recruitment of myeloid-derived suppressor cells via the inhibition of IL-17 expression in γδT17 cells [9]. Such studies could show the potential of CA as an antineoplastic drug, but the active ingredients and the exact mechanism of CA for cancer treatment have not been fully elucidated.

According to the “disease-target protein-drug” network, network pharmacology could explore the anti-disease mechanisms of drugs more systematically and comprehensively for guiding drug development and disease research [10]. The application of molecular docking in modern medical research could discover and predict the ligand conformation on binding sites between protein macromolecules and targets. At the same time, this technique also utilizes key processes involved in molecular recognition to calculate the affinity between ligands and receptors [11]. Some studies used the method of network pharmacology to explore the potential therapeutic targets and biological mechanisms of CA in the treatment of fibrotic disorders, but only one or six compounds were involved [12,13,14]. In addition, through the combination of network pharmacological prediction and experimental verification, it was reported in the literature that CA can improve cardiac hypertrophy, hypertrophic scar, and liver injury [15,16,17]. The study of network pharmacology regarding CA involved very few components and has not yet been applied to an anti-angiogenesis activity. In our study, more active components of CA were screened out based on the drug-like parameter.

In a previous report, Lusardi et al. illuminated the correlation between anti-angiogenic activity and the signaling pathways involving AKT and VEGF [18]. Additionally, there are reports indicating that asiatic acid can inhibit VEGF to accomplish anti-angiogenesis [19]. Confoundingly, in studies on wound healing, compounds like asiaticoside have been found to enhance the expression of VEGF in the skin [20,21]. However, the anti-angiogenic capabilities of analogous compounds remain unexplored. To enhance the exploration of anti-angiogenic efficacy in cancer research, characteristic components of CA, triterpenoids, were systematically validated using network pharmacology and experimental validation. This involved identifying potential targets, establishing a VEGF-stimulated model for vascular endothelial cells, and a comparison of efficacy differences between extracts and single compounds. This research aims to offer evidence that deepens our theoretical insights into the active ingredients and mechanisms through which CA addresses cancer and explore its potential clinical applications.

## 2. Results

### 2.1. Network Pharmacology

#### 2.1.1. Targets of CA and Angiogenesis

After removing the repeated compounds, a total of 39 components of CA were obtained from the TCMSP database. In total, 422 potential targets of active compounds of CA were discovered from the TCMSP database and SwissTargetPrediction database. The keyword “angiogenesis” was used to search relevant targets in the GeneCards, DrugBank, OMIM, and TTD databases, respectively, and a total of 1646 angiogenesis-related targets were found.

#### 2.1.2. Common Target Acquisition and PPI Network Construction

**Figure 1:**
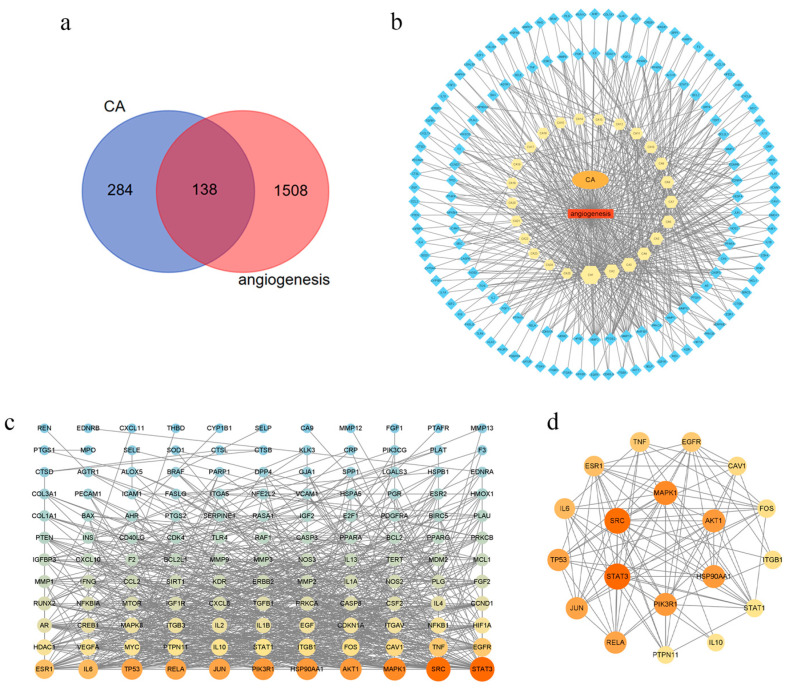
CA and angiogenesis-related targets, disease–drug–pathway–target network, and PPI network in colon cancer treatment. (**a**) Venn diagram of potential targets for the anti-angiogenesis of CA; (**b**) compound–target network of CA for anti-angiogenesis; (**c**) PPI network of CA for anti-angiogenesis; (**d**) core target of PPI network.

Then, 138 common targets between CA and angiogenesis were selected which were the potential targets of CA applied to anti-angiogenesis; a Venn diagram is shown in Figure 1a. The PubChem IDs and molecular formulae of the 25 components of CA related to anti-angiogenesis were confirmed in the PubChem database (Table 1). The target and compound information was imported into Cytoscape (version 3.8.2) to obtain a visual network compound–target diagram for CA for anti-angiogenesis (Figure 1b) in which the yellow hexagonal nodes represent the active components of CA and the blue diamond nodes represent the targets.

When the 138 targets were uploaded to the String database, a PPI network was obtained which had 132 nodes and 843 edges, and the non-interacting target proteins were removed. The parameters of the target were analyzed using the tool “Analyze Network”. The average degree value of the node is 12.68. Visualization of the PPI network showed that the larger the shape and the brighter the color, the greater the degree value of the node (Figure 1c).

#### 2.1.3. Core Targets Analysis

The data obtained in “Section 2.1.2” were re-selected using the condition of degree ≥ 23 betweenness centrality ≥ 0.011982 and closeness centrality ≥ 0.444444. According to the degree value, nineteen core targets of CA in the treatment of angiogenesis, such as signal transducer and activator of transcription 3 (STAT3), SRC, mitogen-activated protein kinase 1 (MAPK1), AKT1, PIK3R1, and HSP90AA1 (Figure 1d), were obtained.

#### 2.1.4. GO and KEGG Enrichment Analyses

**Figure 2:**
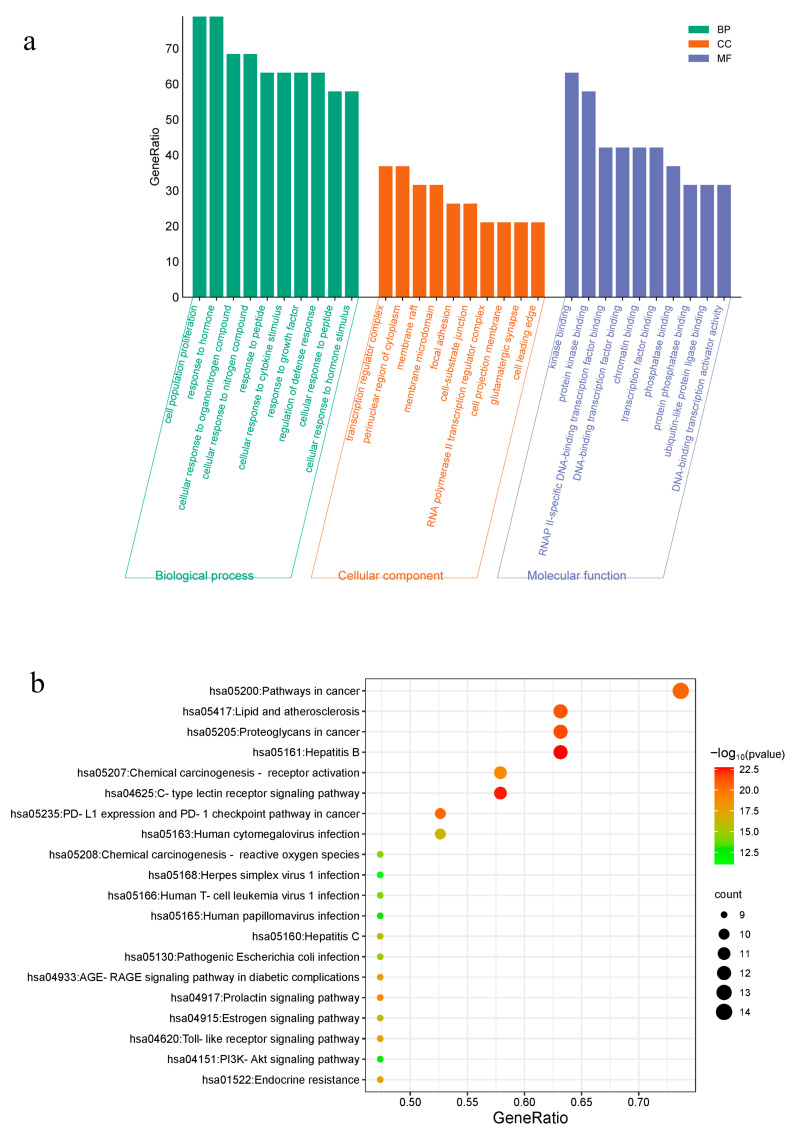
Analysis of GO and KEGG enrichment. (**a**) Top 10 GO terms in the biological process (BP), cellular component (CC), and molecular function (MF) categories (*p* < 0.05); (**b**) top 20 KEGG pathways (*p* < 0.05).

The above 19 core targets were analyzed using a Metascape analysis tool for GO and KEGG. The results of the GO enrichment analysis showed that there were 630 BP, 30 CC, and 53 MF. The first 10 were displayed according to GeneRatio (Figure 2a), mainly including cell population proliferation, transcription regulator complex, kinase binding, and so on.

The KEGG enrichment analysis output 141 signal pathways. According to the GeneRatio values, the most significant 20 pathways linked to the anti-angiogenesis effect of CA were selected to draw the bubble chart (Figure 2b). It was noteworthy that a large proportion of genes were involved in cancer-related pathways such as programmed cell death protein 1 (PD-1), AGE-RAGE, Toll-like receptor (TLR), and PI3K-Akt.

#### 2.1.5. Molecular Docking Analysis

**Figure 3:**
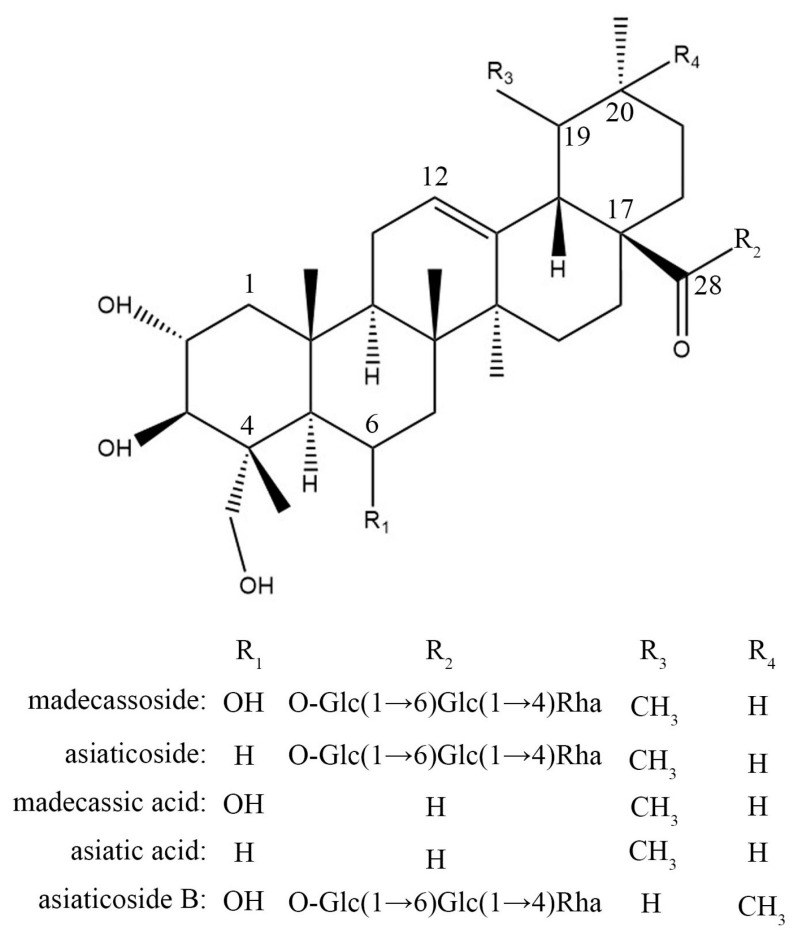
The structure of five core components from CA.

**Figure 4:**
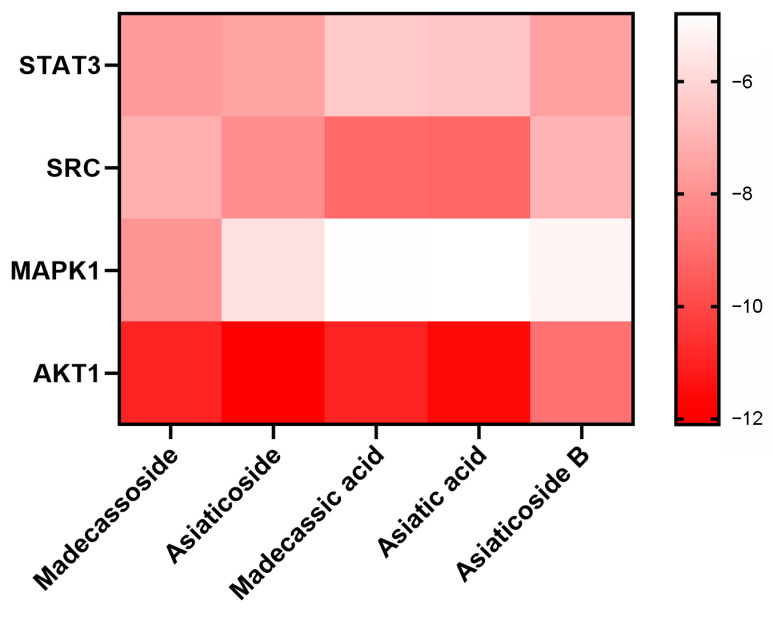
A heat map of binding energies between important active components of CA and the core targets of angiogenesis.

In order to verify the binding energy between the angiogenesis-related targets and active components in CA, STAT3 (PDB: 6NJS), SRC (PDB: 2H8H), MAPK1 (PDB: 5LCJ), and AKT1 (PDB: 6S9W) were selected as receptors, and the characteristic components of CA (madecassoside, asiaticoside, madecassic acid, asiatic acid, and asiaticoside B) were considered ligands for molecular docking, and their structures are shown in Figure 3. A binding energy < 0 indicates that the two molecules could bind spontaneously. The smaller the binding energy is, the stronger the molecular binding ability is and the more stable the conformation is. Binding energy results showed that the affinity of SRC, AKT1, and STAT3 combined with all ligands was less than −5.5 kcal/mol, indicating their great binding. In addition, the binding energy between madecassoside and each receptor was less than −7.0 kcal/mol showing the strong affinity (Figure 4). Thus, the active components of CA in the treatment of angiogenesis have great binding activity to protein targets, and the prediction results are credible.

These five compounds have a common parent nucleus called pentacyclic triterpene. The o-glycosylation of triterpenes at position C28 exhibited better binding activity on STAT3; for SRC, the o-glycosylation at position C28 and the hydroxylation at position C6 of the triterpenes could weaken its binding activity; The o-glycosylation at position C28, the methylation at position C19, and the hydroxylation at position C6 were beneficial for the binding activity with MAPK1. The unhydroxylated forms at position C6 and the methylation at position C19 had a great influence on the binding activity of AKT1. Therefore, ursane-type triterpenoids might possess a better binding mode and affinity when interacting with angiogenesis-related targets, resulting in a significant difference in inhibitory activity.

### 2.2. Experimental Outcomes

#### 2.2.1. Cellular Anti-Proliferation Activity

**Figure 5:**
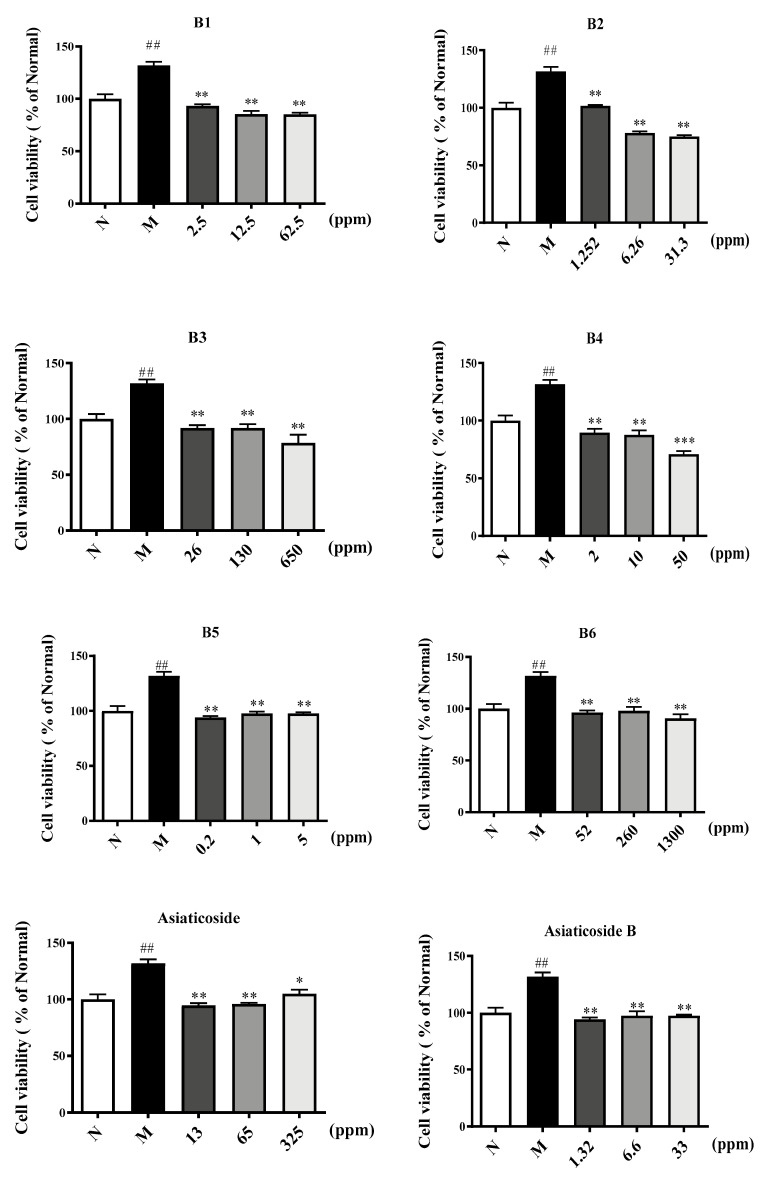

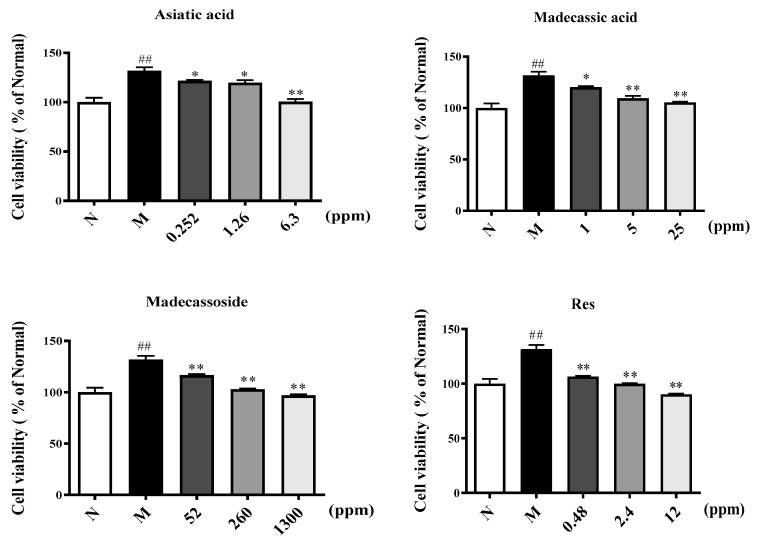
Six extracts (**B1**–**B6**) and five core components from CA inhibit the proliferation of HUVECs compared to resveratrol as a positive control. The *p*-value, ## *p* ≤ 0.01 in contrast to NS group, * *p* ≤ 0.05, ** *p* ≤ 0.01 and *** *p* ≤ 0.001 compared to Model group.

To further determine whether the anti-cancer effect of CA in vivo depends on its anti-angiogenesis effect, its function on the proliferation of HUVECs was examined via an MTT assay. As presented in Figure 5, six kinds of CA extracts significantly inhibited HUVEC proliferation compared with the model group at 24 h, and the inhibitory effect of five important components of CA on HUVEC proliferation was also obvious. These results illustrate that the suppression of vascular endothelial cell proliferation is perhaps important in the anti-cancer process of CA. A comparison of the five components from CA showed significant inhibitory activity on cellular proliferation that was due to a lack of the o-glycosylation at position C28, methylation at position C19, and hydroxylation at position C6.

#### 2.2.2. The Cellular Anti-Migration Activity of Components from CA

**Figure 6:**
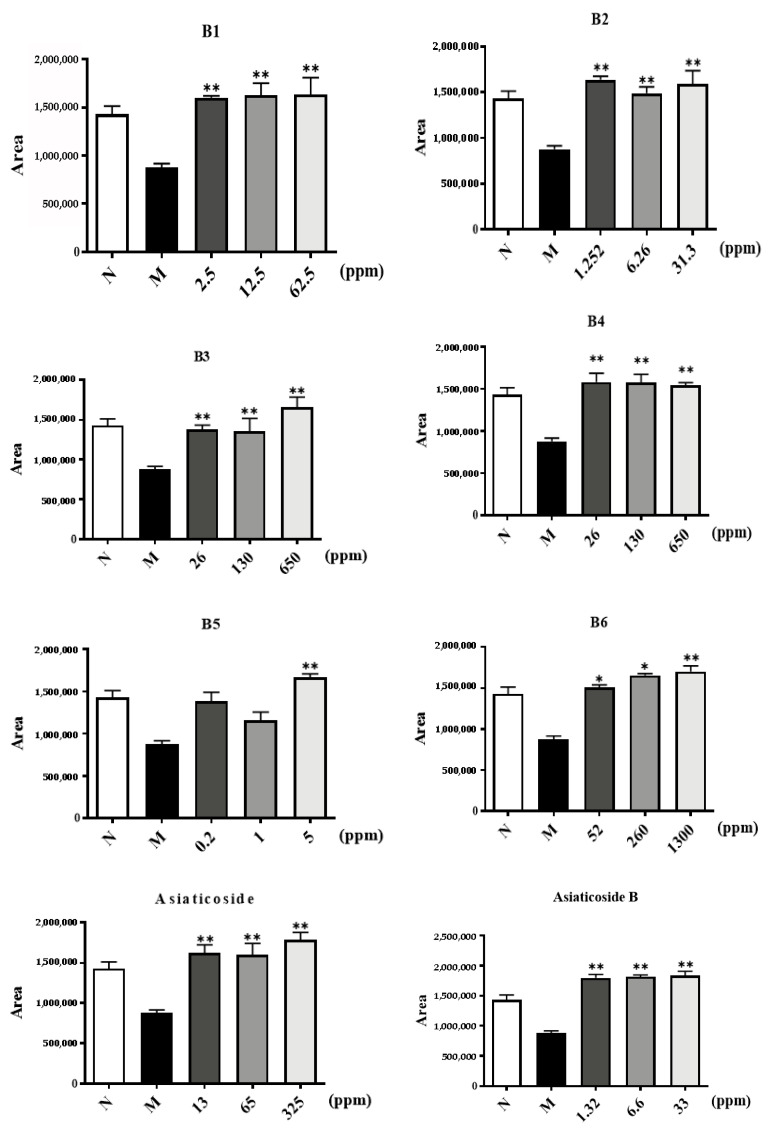

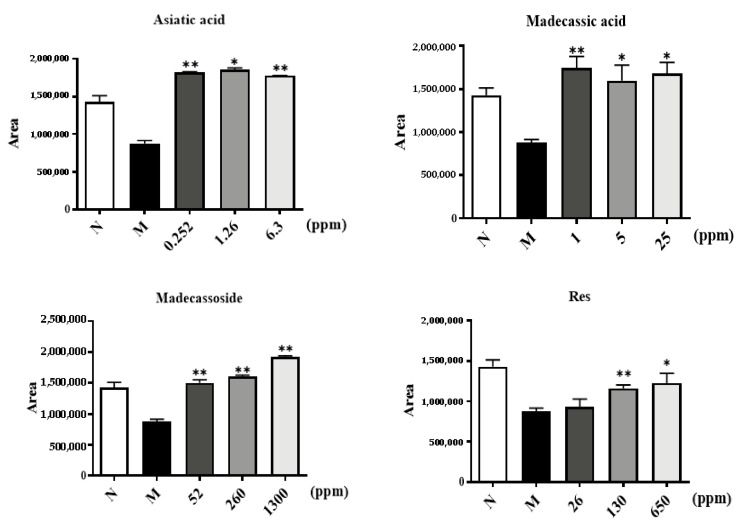
Six extracts (**B1**–**B6**) and five core components from CA inhibit the migration of HUVECs compared to resveratrol as a positive control. The *p*-value, * *p* ≤ 0.05, and ** *p* ≤ 0.01 compared to Model group.

To investigate the effect of components from CA on the migration of HUVECs, a wound-healing assay was performed. As presented in Figure 6, the wound-healing capacity of HUVECs was diminished by the extracts and components of CA at 8 h after the wounds were created. Figure S1 shows original experimental information on the wound-healing assay. According to a quantitative comparison of the cell migration area, the migration of HUVECs was inhibited by B6 and madecassoside in a dose-dependent manner. The absence of the o-glycosylation at position C28, the methylation at position C19, and the hydroxylation at position C6, seemed to increase the inhibitory potential of the cellular migration.

#### 2.2.3. The Cellular Anti-Vascular Tube Formation Activity

**Figure 7:**
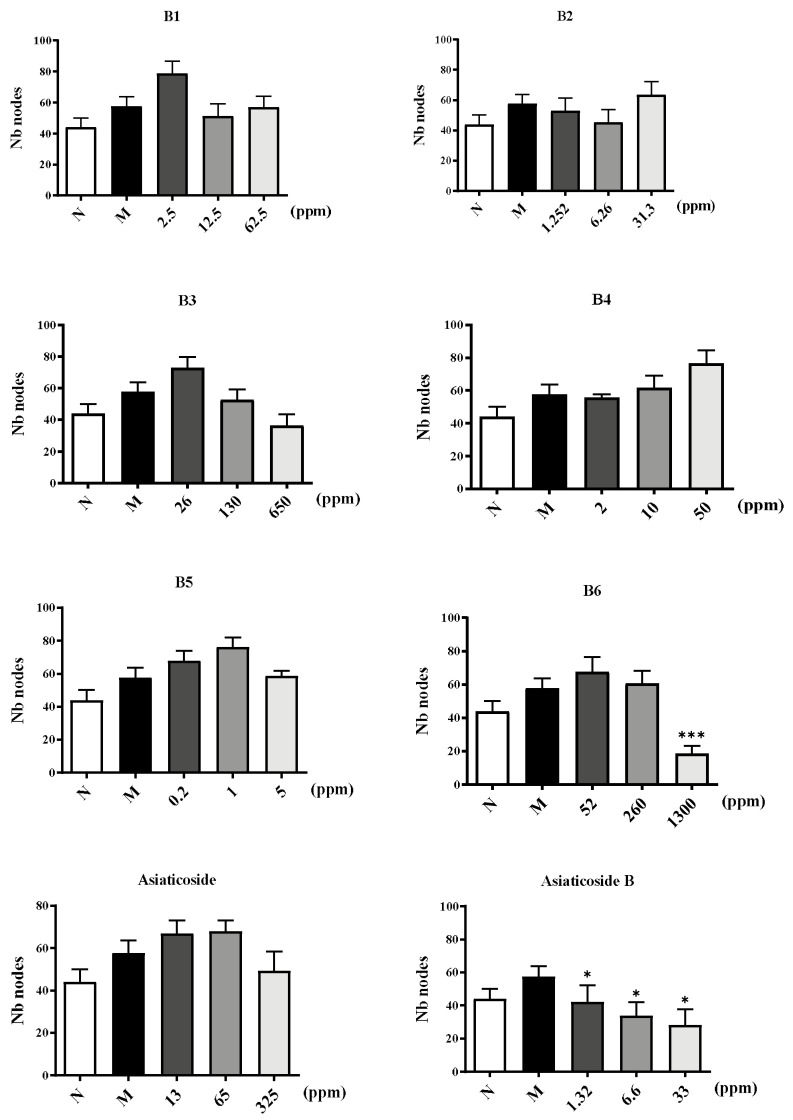

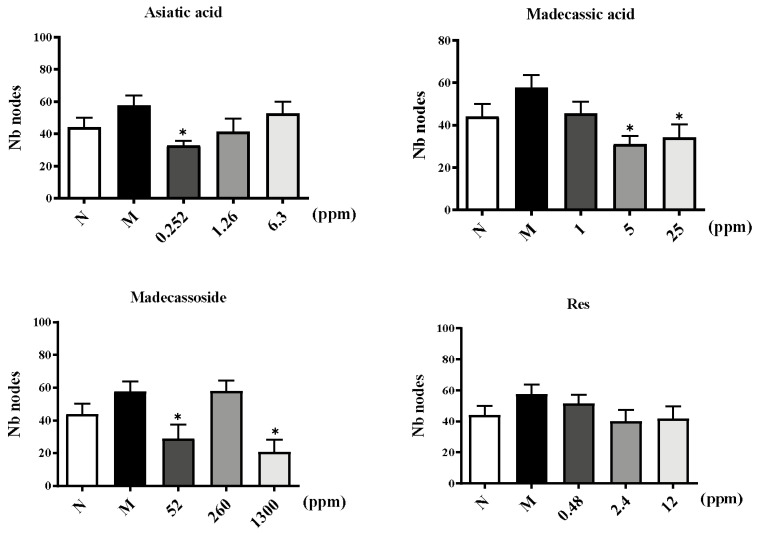
Six extracts (**B1**–**B6**) and five core components from CA inhibit the node formation of HUVECs compared to resveratrol as a positive control. The *p*-value, * *p* ≤ 0.05, and *** *p* ≤ 0.001 compared to Model group.

**Figure 8:**
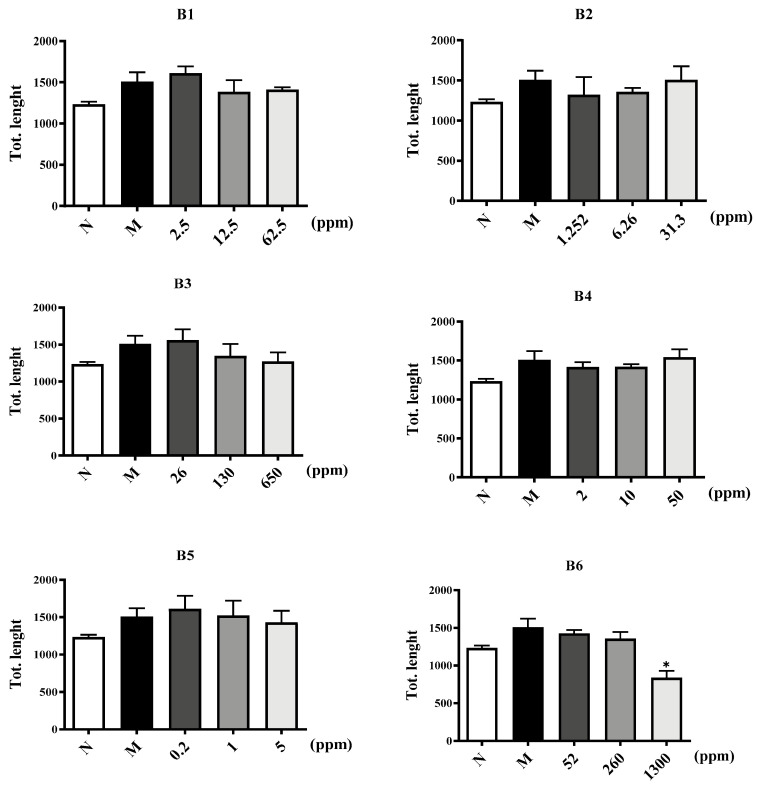

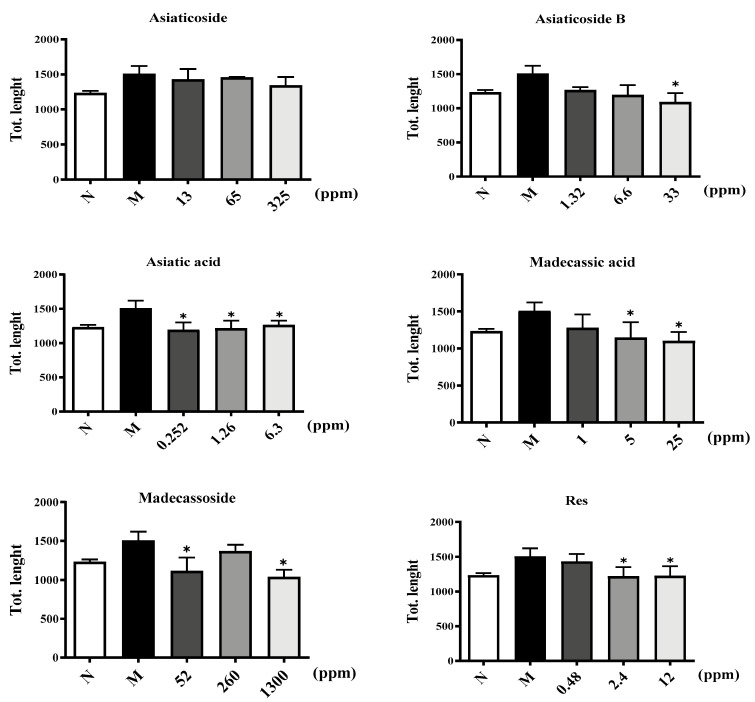
Six extracts (**B1**–**B6**) and five core components from CA inhibit the tube formation of HUVECs compared to resveratrol as a positive control. The *p*-value, * *p* ≤ 0.05, compared to Model group.

Subsequently, whether components of CA were able to disrupt endothelial network formation was investigated using a Matrigel assay. HUVECs were plated onto Matrigel in the presence of VEGF165 as angiogenic factors. Then, the cells were treated with components of CA in different concentrations or NS as a control for 8 h, and microphotographs were then obtained. Pictures of the cords of interconnecting cells were generated using ImageJ software (version 1.52a), and the number of nodes and the length of tube formations were quantitatively analyzed in Figure 7 and Figure 8. Figure S2 shows the original experimental information from the Matrigel assay. Significant decreases in tube formation in the B6, asiaticoside B, madecassoside, asiatic acid, and madecassic acid groups were observed where normal tube structures were destroyed with interrupted alignments and cords. The tube structures observed in other groups were as regular as those in the control group. The deficiency of o-glycosylation at position C28 and the methylation at position C19 of the triterpenoids may play a crucial role in inhibiting vascular tube formation.

## 3. Discussion

*Centella asiatica* (CA) is a TCM with extensive medicinal value which was commonly used in Southeast Asian countries. CA and its main components have an effect on neurological, endocrine, skin, cardiovascular, gastrointestinal, immune, and gynecological diseases. The underlying mechanisms of CA’s action are quite diverse [22], for instance, CA has anti-inflammatory, anti-oxidative stress, and anti-apoptotic effects and causes improvements in mitochondrial function. Among the effects, many studies focused on anticancer effects and showed remarkably inhibitory effects on many kinds of cancers, including liver cancer, colon cancer [23], and breast cancer [24].

Tumor blood vessels play a significant role in tumor growth and metastasis. Vascular endothelial cells are an important component of blood vessels as they do not undergo transformation and are less prone to developing drug resistance [19]. The development of effective and low-toxicity anticancer drugs by targeting vascular endothelial cells has important clinical significance for malignant tumor prevention and treatment. In the present study, through network pharmacology and in vitro experiments, we demonstrated for the first time that the pharmacological mechanisms of CA and its triterpenes act on multiple targets and signal pathways, and inhibit HUVECs proliferation, migration, and tube formation, suggesting that it has the potential to become a novel treatment for cancer by targeting angiogenesis.

Combined with network pharmacology and molecular docking, relevant databases and software were used to explore the mechanism of CA in the treatment of angiogenesis. The network of drug ingredients–disease targets showed that there were as many as 25 bioactive components related to the anti-angiogenesis function in CA, including quercetin, apigenin, ursolic acid, madecassoside, asiaticoside, madecassic acid, asiatic acid, and asiaticoside B, verifying the characteristics of the multiples components and targets of traditional Chinese medicine. Nineteen core target proteins were screened by PPI network relationship, among which STAT3, SRC, MAPK1, AKT1, PIK3R1, and HSP90AA1 were located in the center of protein interaction. A KEGG enrichment analysis showed that the key targets of CA in the prevention and treatment of angiogenesis were mainly pathways in cancer: the PD-1, AGE-RAGE, TLR, and PI3K-Akt pathways, and so on. Molecular docking experiments showed that there is a high level of binding activity between the active components of CA and the key targets.

Signal transducer and activator of transcription 3 (STAT3) is a critical transcription activator in angiogenesis; it has been reported that the JAK2/STAT3 and mTOR/STAT3 signaling pathways promote apoptosis and inhibit proliferation and angiogenesis in colorectal cancer cells [25]. Steroid receptor coactivator (SRC) is an intracellular tyrosine kinase so that focal adhesion kinase (FAK) binds to SRC to play a role in tumor angiogenesis. STAT3 is a marker for tumor angiogenesis which interacts with SRC [26]. Furthermore, a study found that microRNA-29b negatively modulates the MAPK/ERK and PI3K/Akt signaling pathways to inhibit angiogenesis in EC by targeting VEGFA [27]. It has been reported that anti-PD-1 combined with endostar has a synergistic effect, dramatically suppressing tumor growth by upregulating cell apoptosis and PI3K/AKT/mTOR-mediated autophagy [28]. The role of AGE-RAGE signaling has been demonstrated in the progression of various types of cancer and other pathological disorders. AGEs-RAGE signaling promotes survival pathways in cancer cells via the negative feedback regulation of apoptosis and the positive regulation of pro-survival mechanisms such as autophagy [29]. TLRs are innate immune receptors involved in the recognition of microbial and self-ligands associated with tissue damage and inflammation. TLR4 is capable of activating the MAPK and nuclear factor kappa-light-chain-enhancer of activated B cells (NF-κB) pathways, implicating the possible direct role of cell-autonomous TLR4 signaling in the regulation of carcinogenesis in particular through the increased proliferation of tumor cells, apoptosis inhibition, and metastasis [30].

Angiogenesis is a multi-step process involving endothelial cell proliferation, migration, tube formation, and matrix reconstruction. To thoroughly investigate the effects of CA and its triterpenes on the HUVECs’ anti-angiogenesis functions, we carried out a series of experiments, finding that B6, asiaticoside B, madecassoside, asiatic acid, and madecassic acid inhibit the VEGF165-induced proliferation, migration, and tube formation of HUVECs.

## 4. Materials and Methods

### 4.1. Network Pharmacology

#### 4.1.1. Identification of Active Components and Potential Targets of CA

The active components of CA were preliminarily obtained by using the traditional Chinese medicine system pharmacology (TCMSP) database according to the drug-like pharmacokinetic parameter, drug-like (DL) ≥ 0.18; other effective ingredients mentioned in the literature were also collected, and the corresponding target proteins were recorded at the same time. Furthermore, the rest of the compounds that did not find the target could be predicted from the SwissTargetPrediction database. All the protein targets were then standardized using the Uniprot Protein Database.

#### 4.1.2. Screening of Potential Target Genes for Angiogenesis

Using “angiogenesis” as the keyword in the GeneCards database, the relevant targets were retrieved and selected by defining the median. Then the angiogenesis-related targets were searched in the DrugBank, OMIM, and TTD databases in turn, and all the targets were merged.

#### 4.1.3. Intersection of Targets for CA and Angiogenesis-Related Targets

The targets of angiogenesis were intersected with the active targets of CA, which represent the potential targets of CA in the treatment of angiogenesis, and a Venn diagram was drawn accordingly. The active components related to angiogenesis in CA were obtained, and their molecular formulas and PubChem IDs were queried from the PubChem database. The target data related to the active components were input into Cytoscape software (version 3.8.2), thus forming a compound–target network of CA for anti-angiogenesis. 

#### 4.1.4. Construction of the Protein–Protein Interaction (PPI) Network

The potential targets were uploaded to the String database to build a protein–protein interaction (PPI) network system, and the data were input into the Cytoscape software (version 3.8.2) to realize the visualization of the results.

#### 4.1.5. Determination of Core Targets

The related parameters of each target protein in the PPI network were analyzed using the tool “Analyze Network” in the Cytoscape software (version 3.8.2); among them, the higher the degree value, the stronger the importance of the target in the network [31]. The basic requirements for screening core targets are that the degree and betweenness centrality value are more than twice the median value and the closeness centrality value is greater than the median value.

#### 4.1.6. GO and KEGG Enrichment Analyses

A GO (gene ontology) analysis classifies genes according to different functions, including biological processes (BPs), molecular function (MFs), and cellular components (CCs) [32]. KEGG (Kyoto Encyclopedia of Genes and Genomes) is a bioinformatics database that summarizes and expounds the related characteristics and pathways of human differentially expressed genes. The selected core targets of CA related to angiogenesis were conducted with GO and KEGG enrichment analyses using Metascape. The data were imported into Origin Lab 2021 for visualization.

#### 4.1.7. Molecular Docking

The significant anti- angiogenesis components of CA were considered ligands, and the selected core targets were used as receptors. The structure of each ligand was drawn using ChemOffice and then treated with energy minimization. The protein structure of the receptor was obtained from the PDB database (https://www.rcsb.org/). The AutoDockVina program was used to realize molecular docking to obtain docking site information and binding energy data. Finally, the docking result was visualized using DiscoveryStudio. The lower the binding energy, the stronger the affinity between the protein receptor and the small molecular ligand. It was generally believed that if the binding energy is lower than −5.5 kcal/mol, then the molecule has good binding activity with the target; if lower than −7.0 kcal/mol, the binding activity is strong.

### 4.2. In Vitro Experiments

#### 4.2.1. Materials

B1–B6 are extracts from CA and differ in the extraction process, which mainly includes various elution stages of over-macroporous resin. Madecassoside, asiaticoside, madecassic acid, asiatic acid, and asiaticoside B are the core components of CA.

#### 4.2.2. Cell Culture and Drug Treatment

The HUVECs were obtained from BeNa Culture Collection (BNCC363216). HUVECs were cultured in an ECM complete medium supplemented with 10% FBS, 1% penicillin, 1% streptomycin, and 1% ECGS in a 5% CO_2_ atmosphere at 37 °C. VEGF165 (vascular endothelial growth factor-165), is a member of the VEGF family that promotes angiogenesis1 by stimulating endothelial cell proliferation and migration [33]. A model group was treated with VEGF165 (200 ng/mL) and the ECM medium (10% FBS, 1% penicillin, and 1% streptomycin). On this basis, the experimental group was incubated with various concentrations of components from CA. The NS group was only cultured with the ECM medium.

#### 4.2.3. Cell Proliferation Assay

Cells (1 × 10^5^ cells/mL) were seeded in 96-well plates and incubated under the aforementioned conditions until 30–50% convergence was reached. Cell treatment was carried out according to the above groups. After an additional 24 h, cell proliferation was determined via an MTT assay. The absorbance of each well at 450 nm was measured. The results were expressed as contrast absorbance, considering the NS group as a control.

#### 4.2.4. Cell Migration Assay

The migration of HUVECs was analyzed using wound assay culture inserts (ibidi, city, Martinsried, Germany). Two-well culture inserts were placed in a 24-well plate. Cells (3 × 10^5^ cells/mL) were seeded in the culture inserts (80 µL/chamber) and divided into four groups, an NS group, a Model group, an Experimental group, and a Positive drug (resveratrol) group, and were incubated with ECM complete medium. When the cells grew to confluency, the silicon inserts were removed, and a homogeneous 500 μm wound was then created (time 0 h). Images were captured after 8 h, and the cell migration capacity was quantified by measuring the area between the wound edges. 

#### 4.2.5. Vascular Tube Formation Assay

Matrigel (Corning) was added to the precooled 96-well plate and left for 45 min. HUVECs (2 × 10^5^ cells/mL) were seeded in culture inserts (ibidi) and divided into four groups: an NS group, a Model group, an Experimental group, and a Positive drug (resveratrol) group. After incubation at 37 °C in 5% CO_2_ for 8 h, images of tube formation were acquired with a light microscope, and the tube length and the number of nodes were analyzed using ImageJ (version 1.52a).

### 4.3. Statistical Analysis

The results were generated from at least three independent experiments and presented as mean ± S.D values. A statistical evaluation was performed using Student’s *t*-test, utilizing the software GraphPad Prism (version 9.0.0). A significant difference was defined as *p* < 0.05.

## 5. Conclusions

In summary, network pharmacology and in vitro experiments provided evidence that the extracts of CA and its triterpenes effectively block angiogenesis. We demonstrated for the first time, that the pharmacological mechanisms of CA and its triterpenes acting on multiple targets and signal pathways and inhibit VEGF-induced HUVECs proliferation, migration, and tube formation. The targets of STAT3, SRC, MAPK1, and AKT1 and the signaling pathways of PD-1, AGE-RAGE, TLR, and PI3K-Akt may be crucial to the anti-angiogenic and anti-cancer activity of CA. Madecassoside, asiaticoside, madecassic acid, asiatic acid, and asiaticoside B of CA are the more highly predictive components. Our findings provide a scientific basis for the study of the complex mechanisms underlying the anti-angiogenic effect of CA, suggesting that it has the potential to become a novel treatment for cancer.

## Figures and Tables

**Table 1:** Twenty-five components of CA with potential anti-angiogenic activity.

Number	Name	Molecular Formula	PubChem ID	Genes Related to Angiogenesis
CA1	Quercetin	C_15_H_10_O_7_	5280343	86
CA2	Apigenin	C_15_H_10_O_5_	5280443	37
CA3	Ursolic acid	C_30_H_48_O_3_	64945	37
CA4	Madecassoside	C_48_H_78_O_20_	45356919	23
CA5	Asiaticoside	C_48_H_78_O_19_	11954171	21
CA6	Madecassic acid	C_30_H_48_O_6_	73412	21
CA7	Asiatic acid	C_30_H_48_O_5_	119034	21
CA8	Asiaticoside B	C_48_H_78_O_20_	91618002	21
CA9	Madasiatic acid	C_30_H_48_O_5_	162998158	19
CA10	Protobassic acid	C_30_H_48_O_6_	21576541	16
CA11	4,5-Dicaffeoylquinic acid	C_25_H_24_O_12_	6474309	14
CA12	3,4-Dicaffeoylquinic acid	C_25_H_24_O_12_	5281780	11
CA13	Chlorogensaure	C_16_H_18_O_9_	12310830	7
CA14	Stachyose	C_24_H_42_O_21_	439531	7
CA15	Neochlorogenic acid	C_16_H_18_O_9_	7067333	7
CA16	Heriguard	C_16_H_18_O_9_	1794427	7
CA17	3,5-Dicaffeoylquinic acid	C_25_H_24_O_12_	6474310	6
CA18	Troxerutin	C_27_H_30_O_16_	5280805	5
CA19	3,5-Dicaffeoyl-4-maloNylquinic acid	C_28_H_26_O_15_	44544976	5
CA20	1,5-Dicaffeoylquinic acid	C_25_H_24_O_12_	6474640	5
CA21	Cynarine	C_25_H_24_O_12_	5281769	4
CA22	Castilliferol	C_24_H_16_O_8_	10526707	2
CA23	Sitosterol	C_29_H_50_O	12303645	1
CA24	Chlorogenic	C_16_H_18_O_9_	94854309	1
CA25	Castillicetin	C_24_H_16_O_10_	102394640	1

## Data Availability

The data presented in this study are available upon request from the corresponding author.

## Supplementary Materials

The following supporting information can be downloaded at https://www.mdpi.com/article/10.3390/molecules29020362/s1, Figure S1: Six extracts (B1–B6) and five core components from CA inhibit the proliferation of HUVECs compared to resveratrol as positive control; Figure S2: Six extracts (B1–B6) and five core components from CA inhibit the vascular tube formation of HUVECs compared to resveratrol as a positive control.

## Abbreviations

CA	*Centella asiatica*
TCM	traditional Chinese medicine
TCMSP	traditional Chinese medicine systems pharmacology
PPI	protein–protein interaction
GO	gene ontology
KEGG	Kyoto Encyclopedia of Genes and Genomes
VEGF	vascular endothelial growth factor
STAT3	signal transducer and activator of transcription 3
MAPK1	mitogen-activated protein kinase 1
PD-1	programmed cell death protein 1
HUVECs	human umbilical vein endothelial cells
CTX	cyclophosphamide
BP	biological process
MF	molecular function
CC	cellular component
DL	drug-like
FBS	fetal bovine serum
FAK	focal adhesion kinase
EC	endometrial carcinoma
NF-κB	nuclear factor kappa-light-chain-enhancer of activated B cells
