# Supplementary material for: Uncovering the Anti-Angiogenic Mechanisms of Centella asiatica via Network Pharmacology and Experimental Validation
Source: Molecules. 2024 Jan 11;29(2):362. doi: 10.3390/molecules29020362 (PMC10821292; doi:10.3390/molecules29020362)
Supplement: Supplementary file 1 [file molecules-29-00362-s001.zip › molecules-2810655-supplementary.pdf]

Supplementary Materials

Uncovering the Anti-Angiogenic Mechanisms of *Centella Asiatica* via Network Pharmacology and Experimental Validation

Bingtian Zhao [1,*], Yuanyuan Li [1], Binya Wang [1], Jing Liu [1], Yang Yang [2,3], Qianghua Quan [2,3], Quan An [2,3], Rong Liang [1], Chunhuan Liu [1] and Cheng Yang [1,*]

[1] Key Laboratory of Synthetic and Biological Colloids, Ministry of Education, School of Chemical and Material Engineering, Jiangnan University, Wuxi 214122, P. R.China; liyuanyuan0918@163.com (Y.L.); dxsxzyzs@163.com (B.W.); lj15735649029@163.com (J.L.); rongliang@jiangnan.edu.cn (R.L.); liuch@jiangnan.edu.cn (C.L.)

[2] Yunnan Baiyao Group Shanghai Science & Technology Co., Ltd., Shanghai, 201100, P. R.China; doubleyoung75@163.com (Y.Y.); 18811781508@163.com (Q.Q.); ynbyanquan@sina.com (Q.A.)

[3] East Asia Skin Health Research Center, Beijing 100037, P. R.China

* Correspondence: btzhao@jiangnan.edu.cn (B.Z.); cyang@jiangnan.edu.cn (C.Y.)

Figure S1. Six extracts (B1-B6) and five core components from CA inhibits the proliferation of HUVECs compared to resveratrol as positive control.

number	A1	A2	A3
Normal	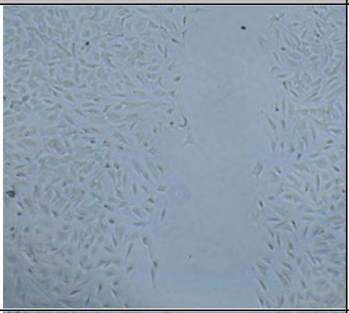	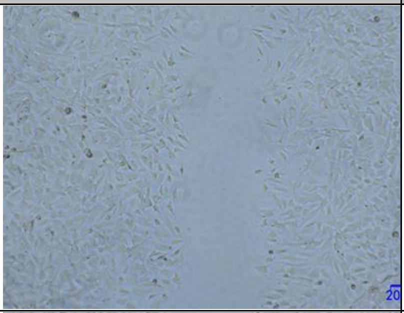	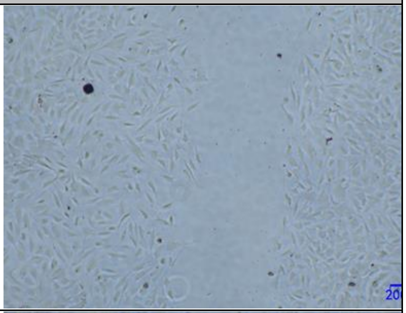
M-VEGF	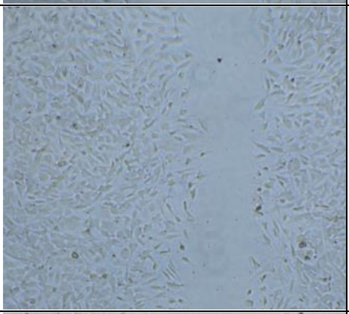	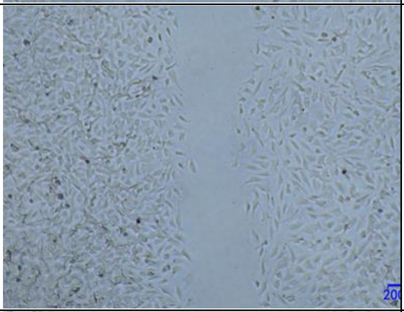	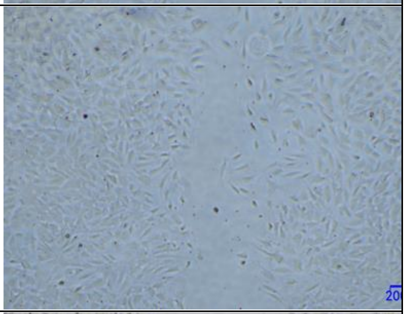
B1-Low	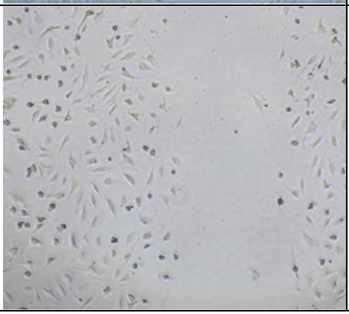	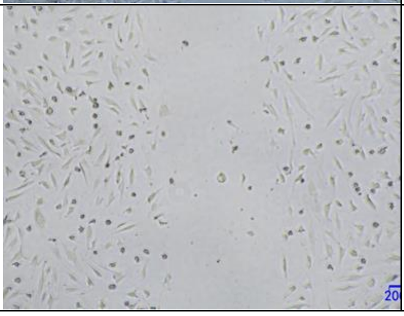	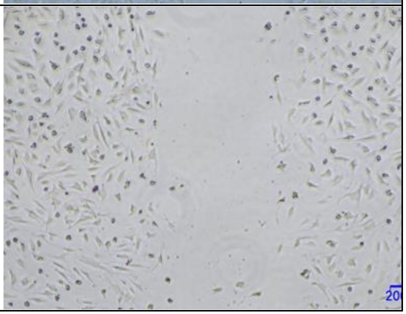

B1-Middle	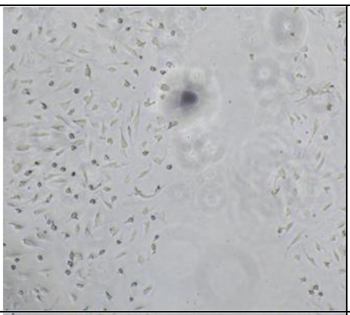	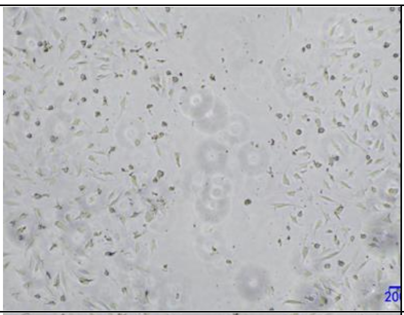	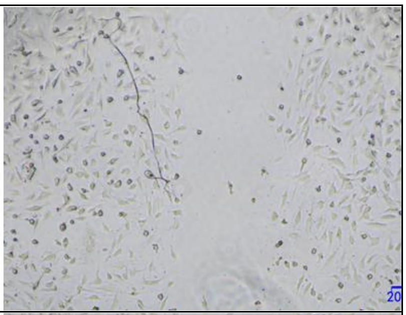
B1-High	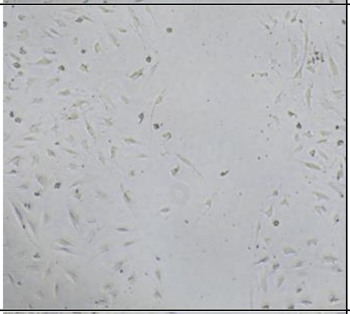	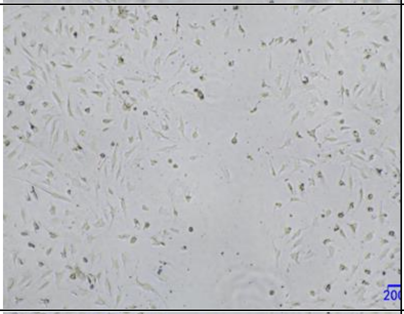	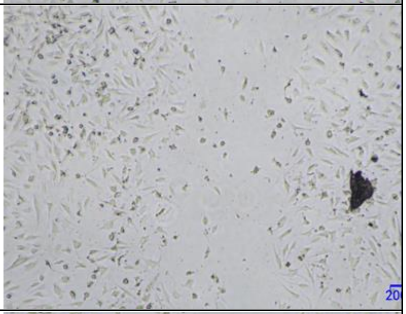
B2-Low	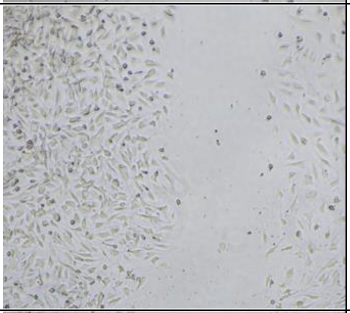	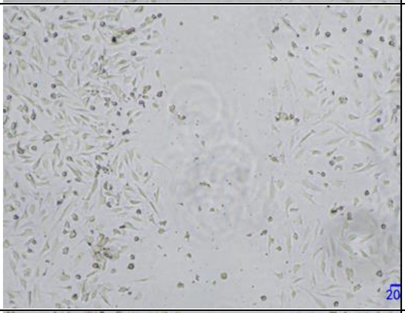	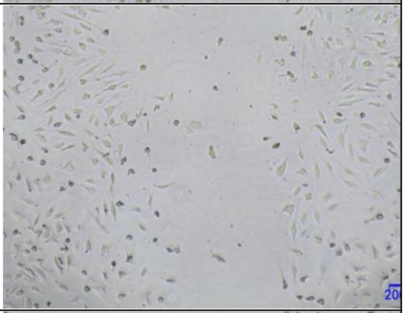
B2-Middle	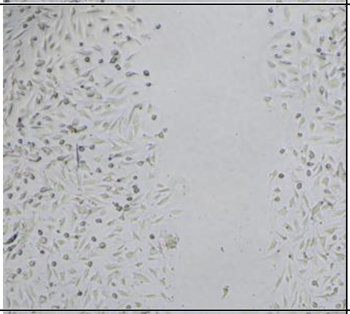	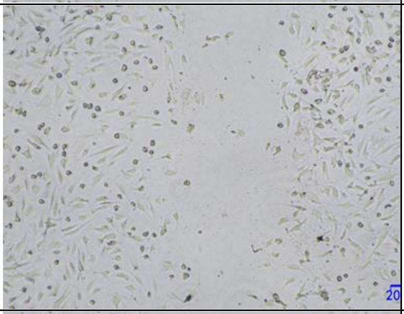	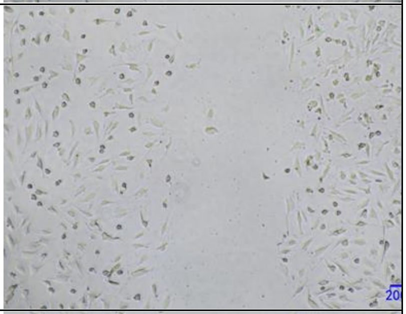
B2-High	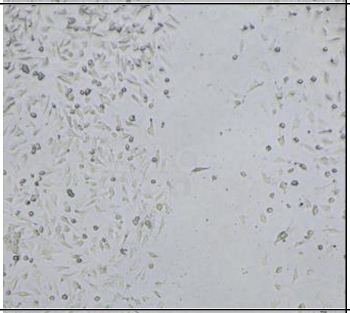	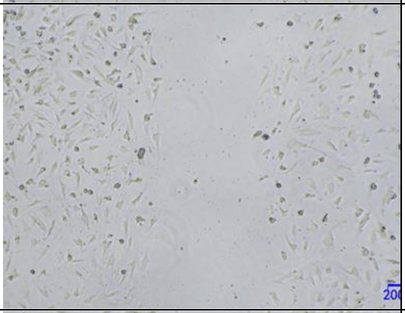	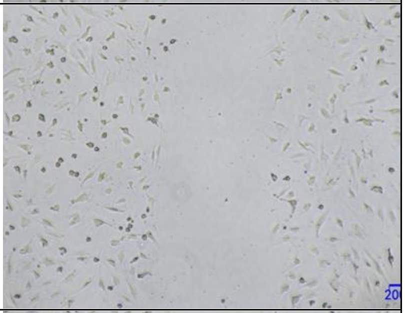
B3-Low	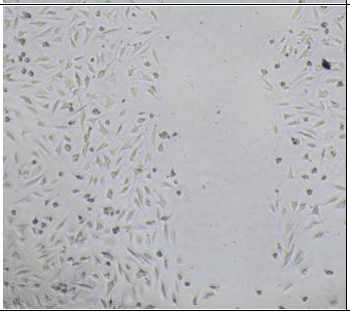	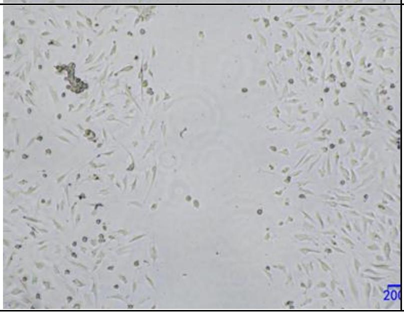	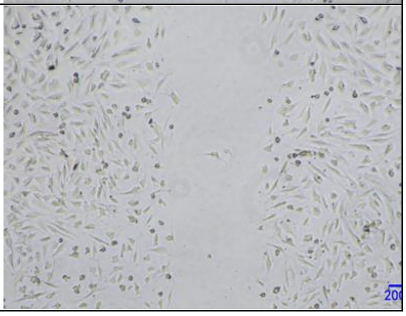

B3-Middle	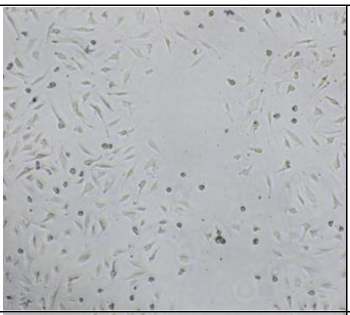	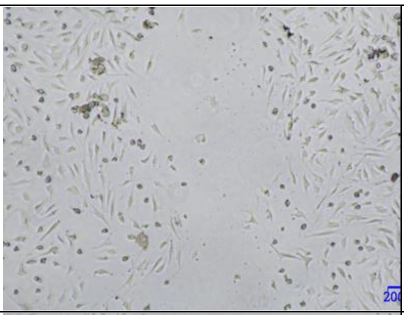	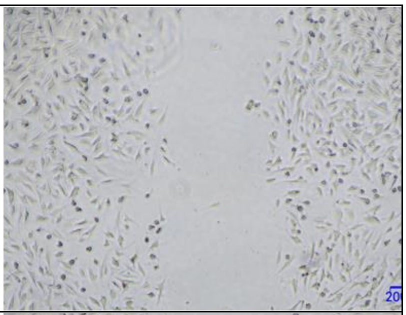
B3-High	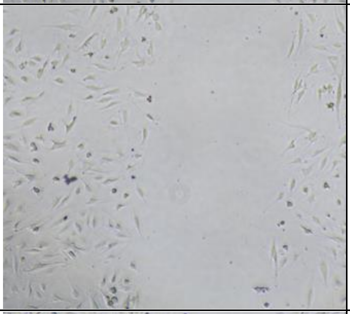	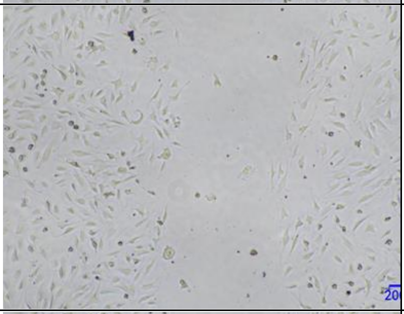	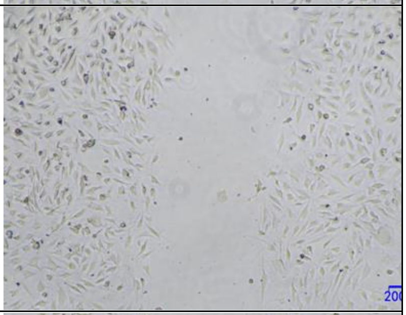
B4-Low	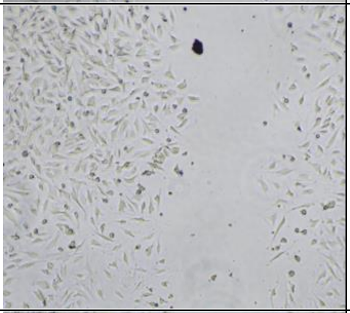	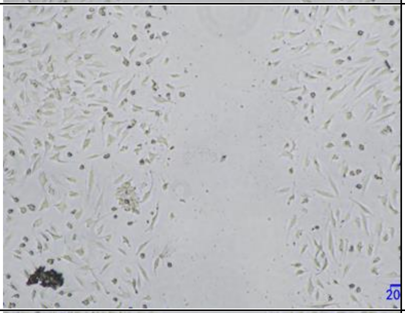	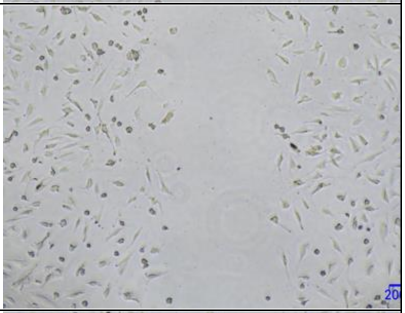
B4-Middle	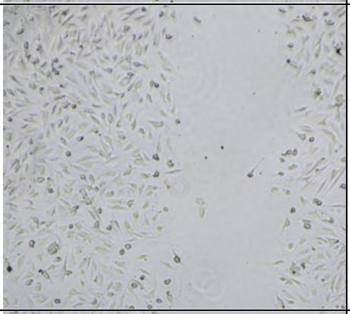	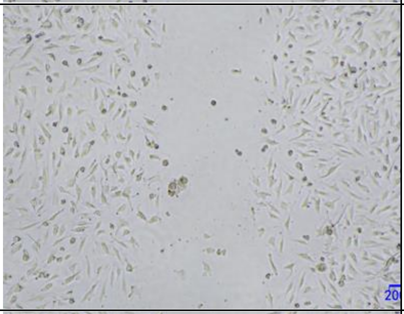	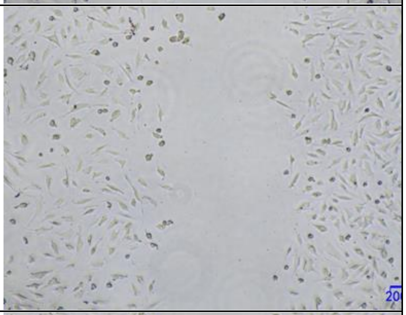
B4-High	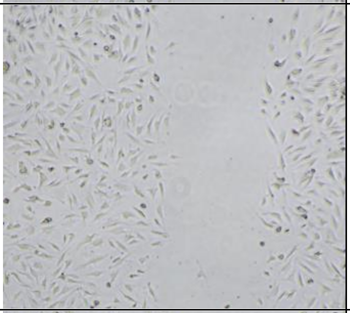	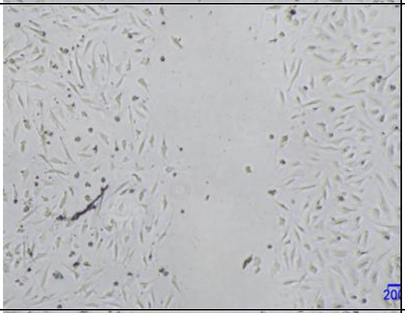	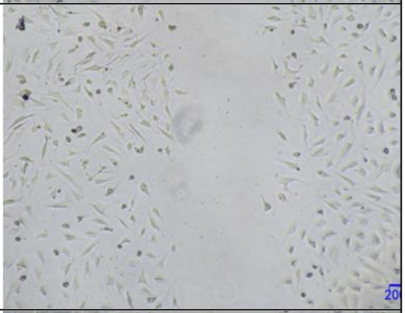
B5-Low	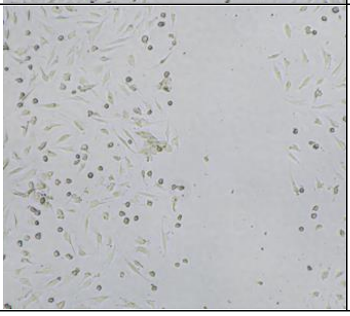	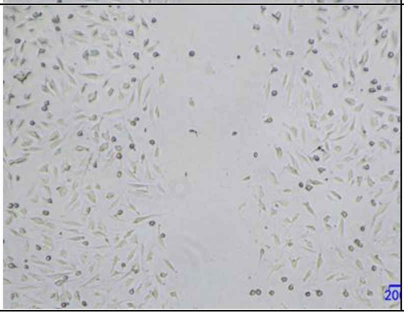	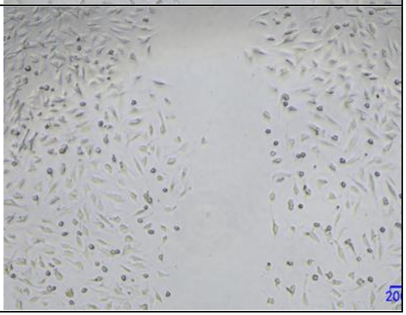

B5-Middle	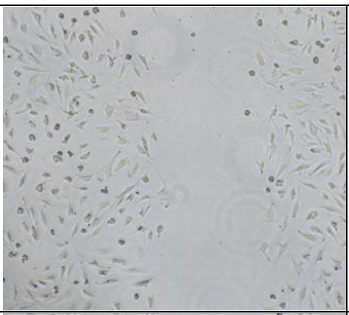	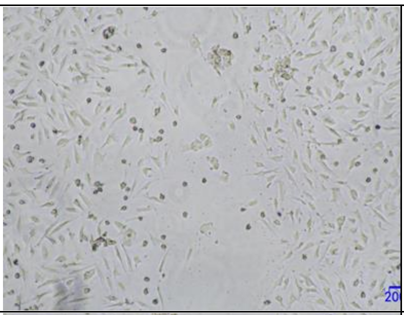	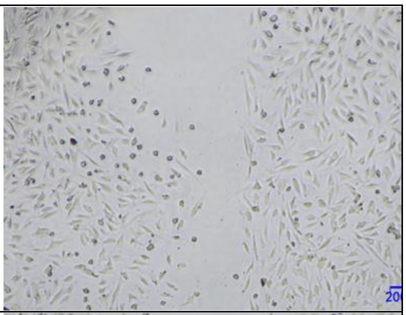
B5-High	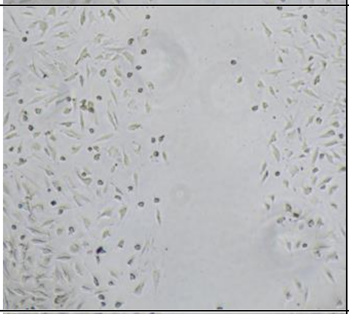	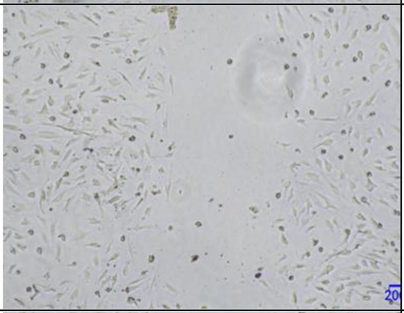	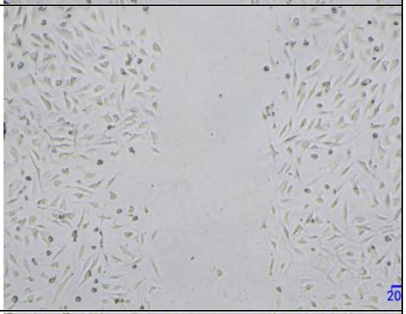
B6-Low	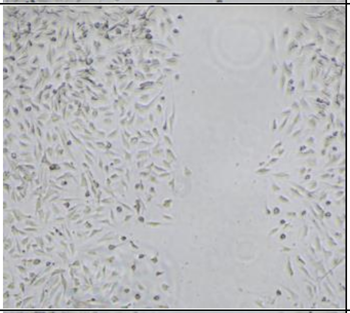	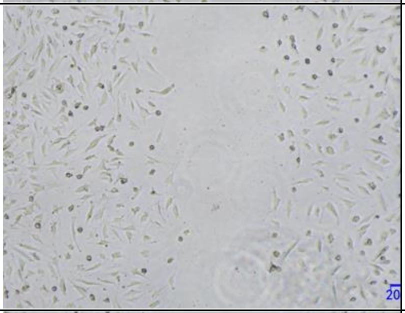	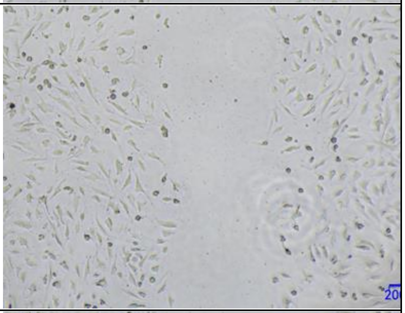
B6-Middle	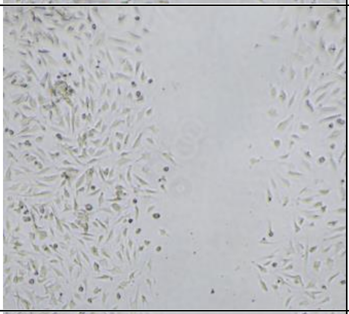	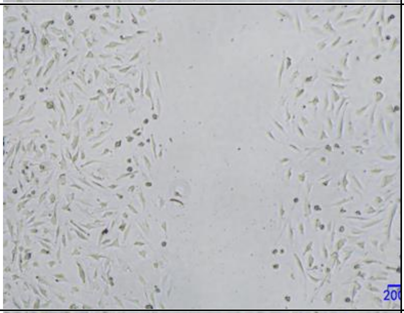	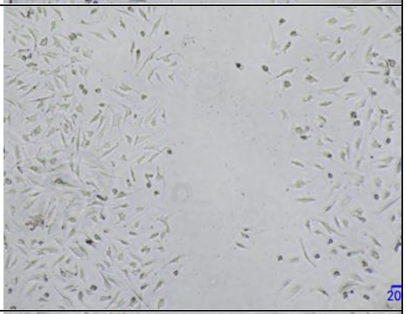
B6-High	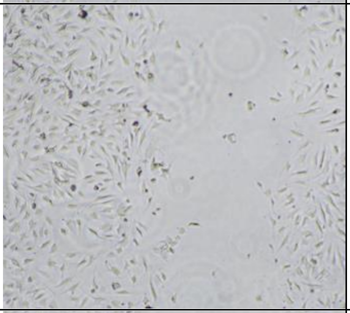	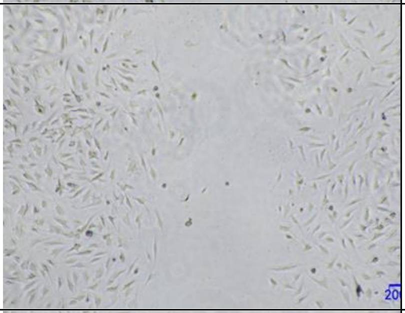	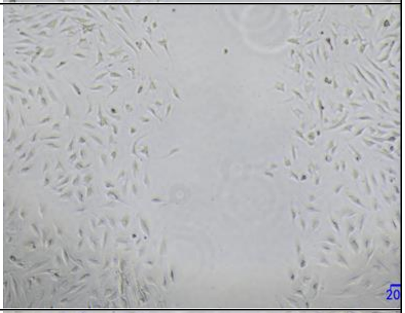
Asiaticoside-Low	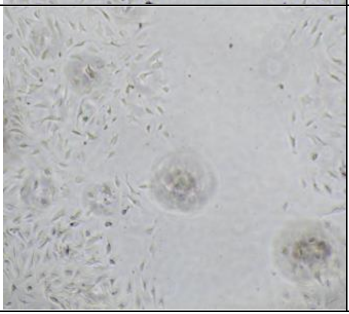	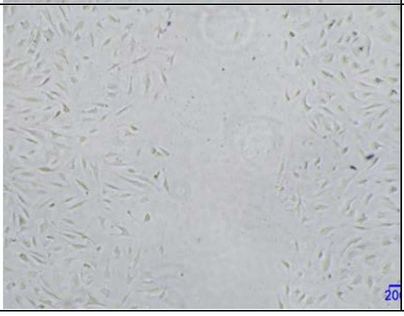	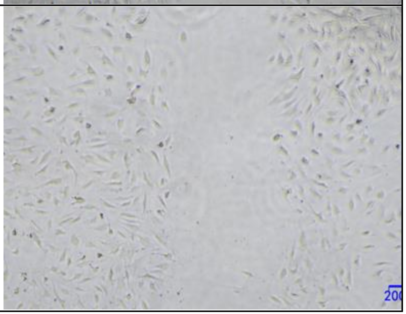

Asiaticoside -Middle	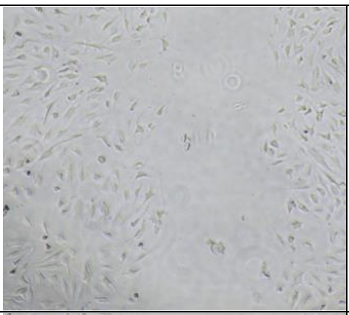	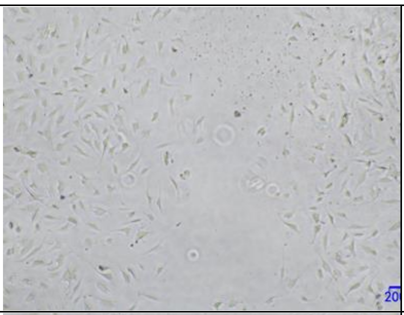	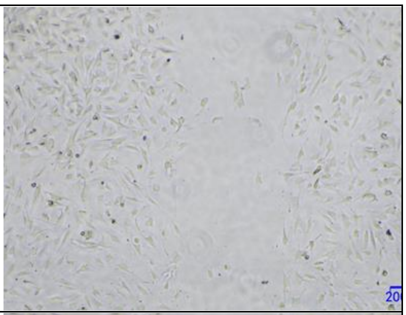
Asiaticoside -High	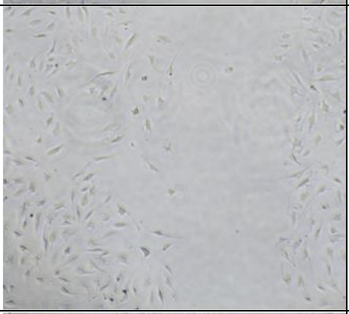	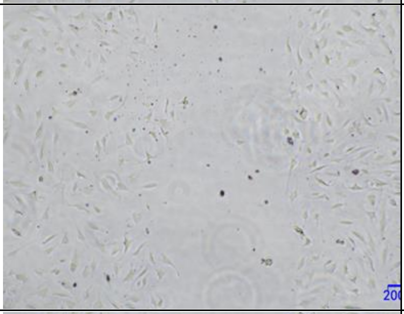	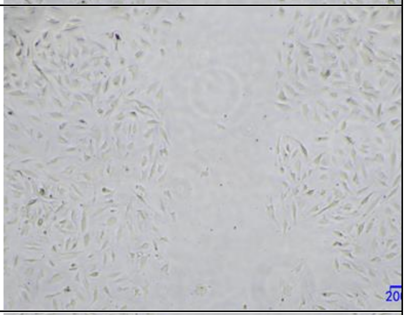
Asiaticoside B -Low	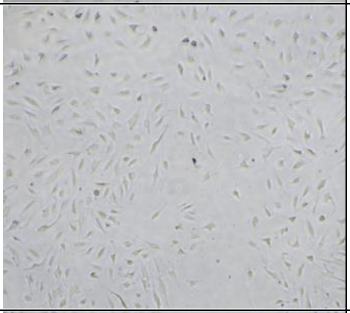	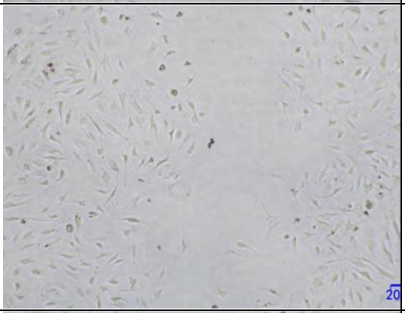	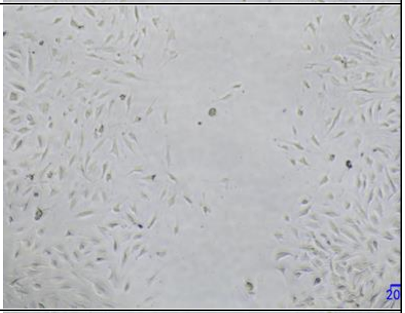
Asiaticoside B -Middle	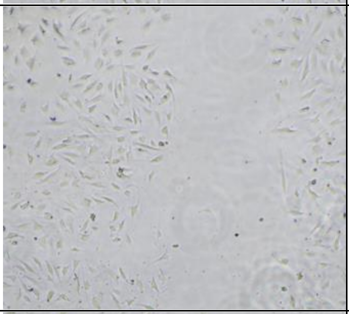	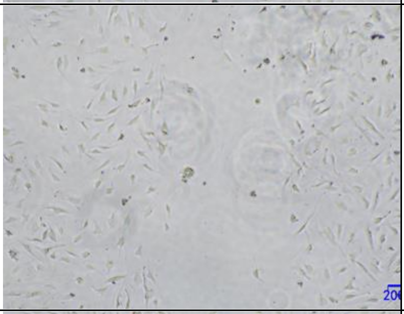	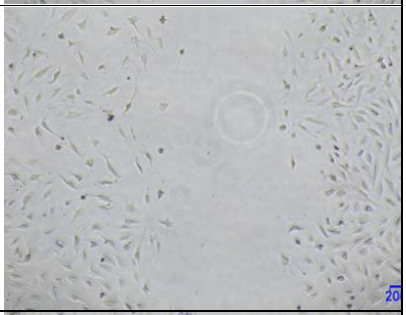
Asiaticoside B -High	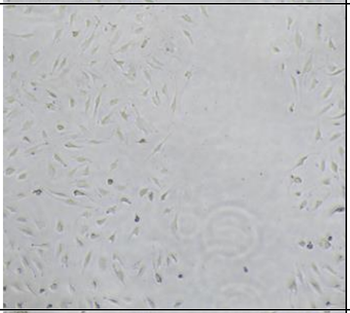	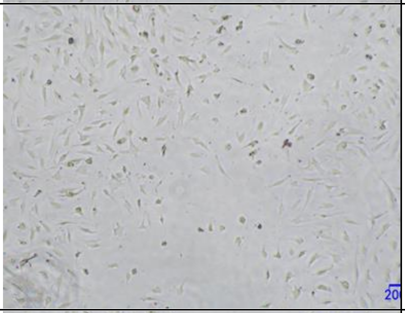	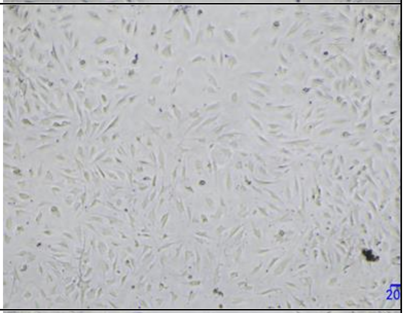
Asiatic acid -Low	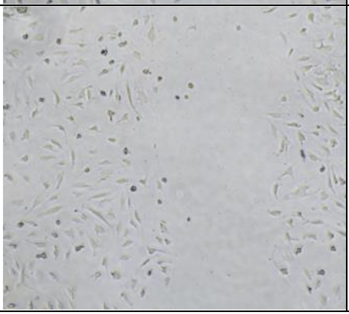	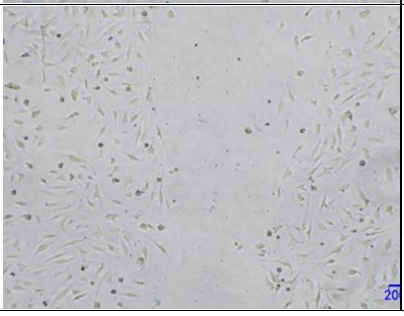	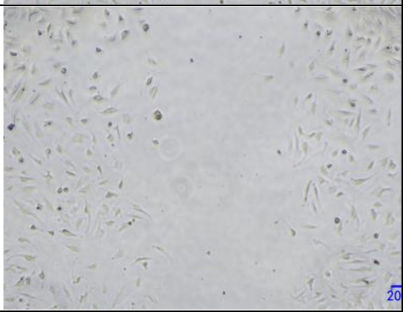

Asiatic acid -Middle	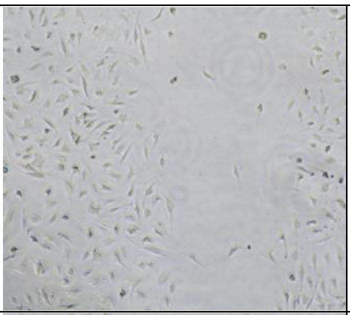	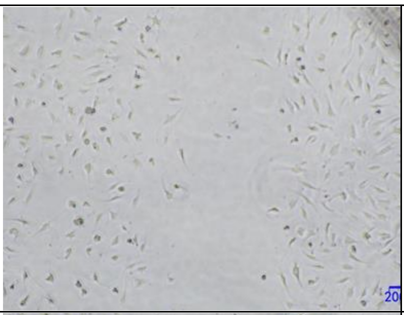	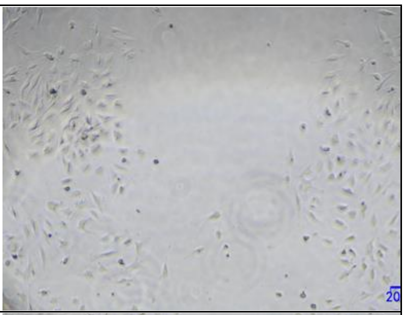
Asiatic acid -High	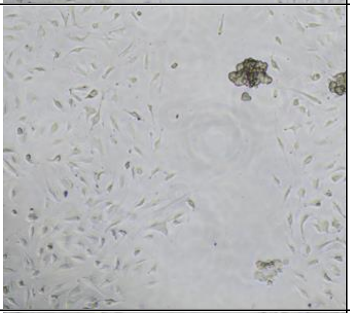	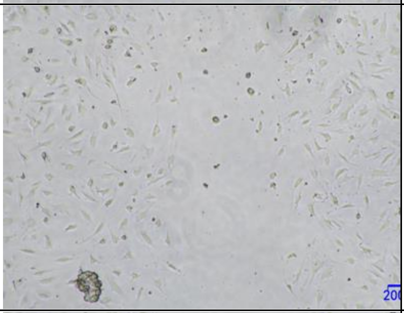	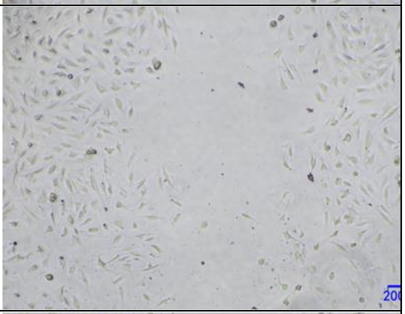
Madecassic acid -Low	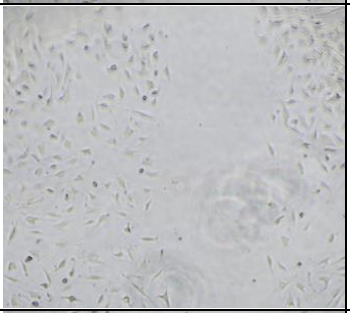	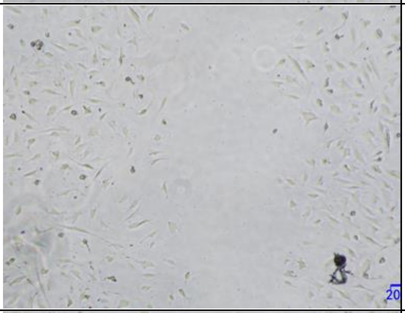	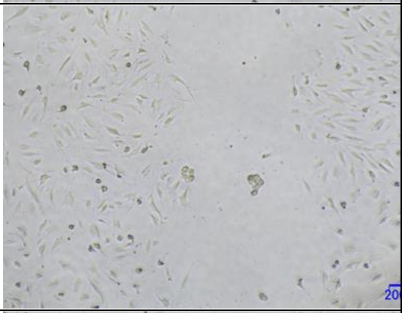
Madecassic acid -Middle	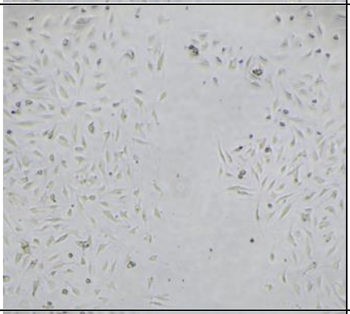	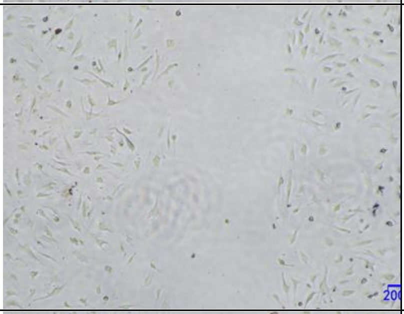	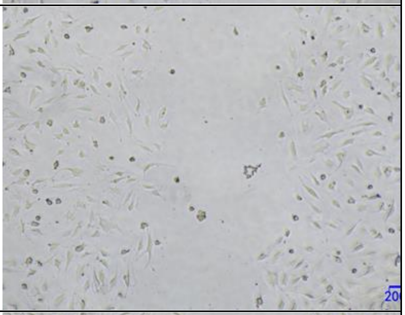
Madecassic acid -High	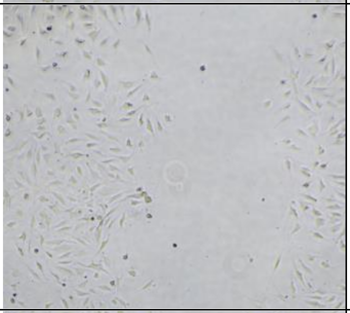	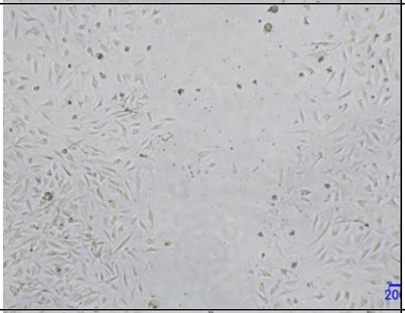	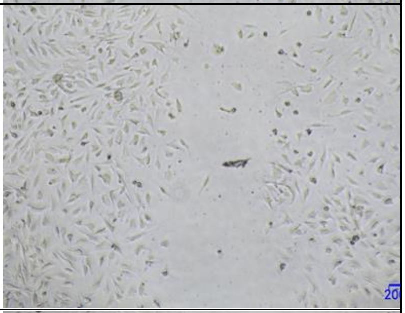
Madecassoside-Low	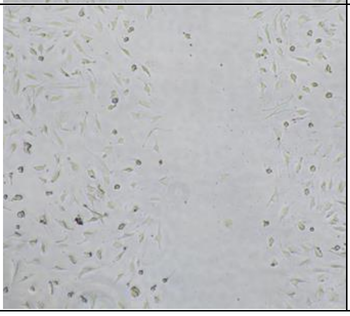	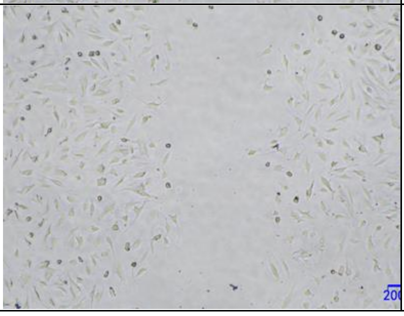	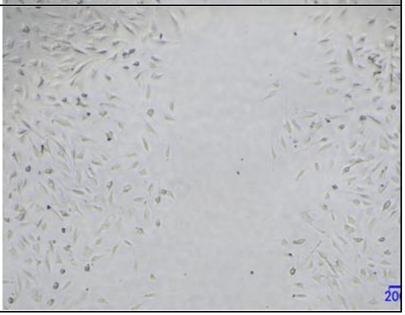

Madecassoside - Middle	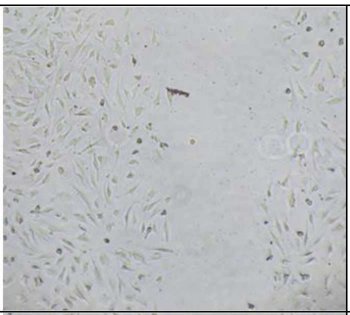	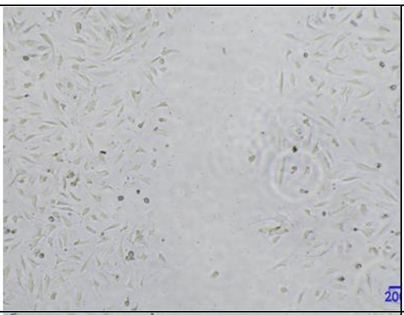	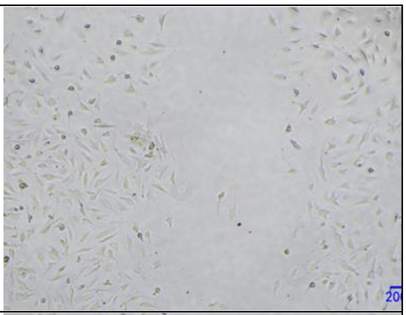
Madecassoside - High	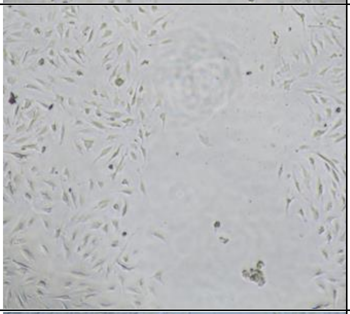	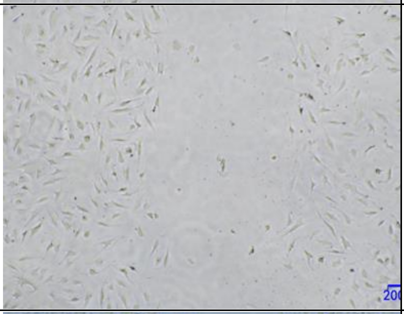	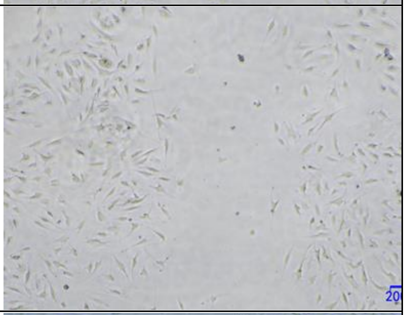
Rev-Low	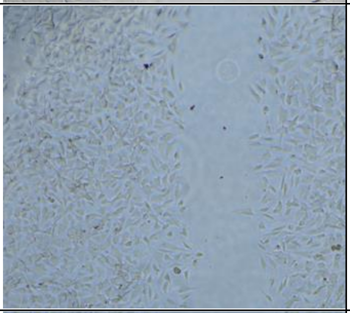	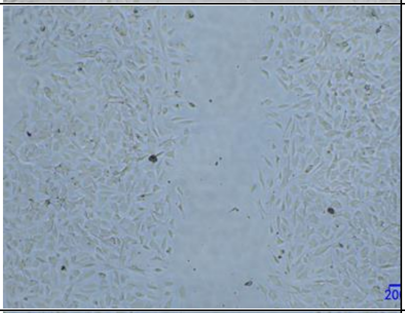	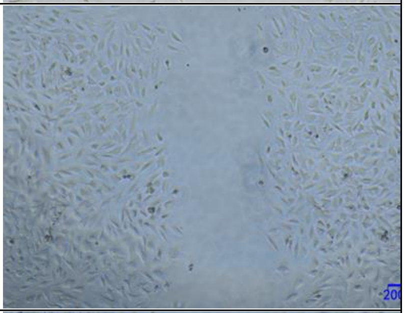
Rev-Middle	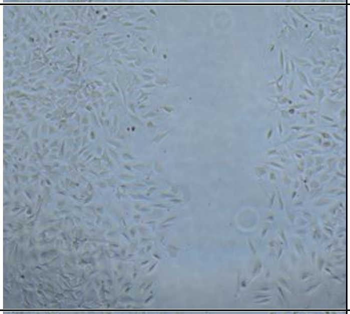	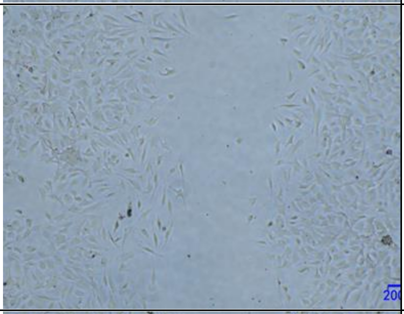	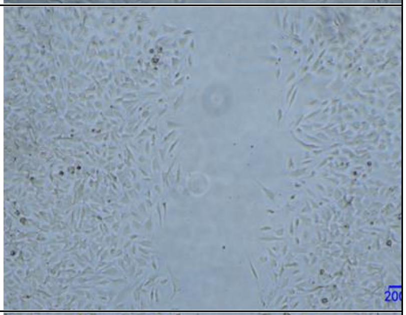
Rev-High	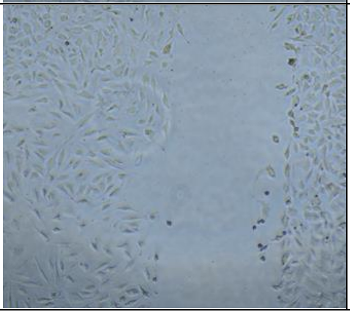	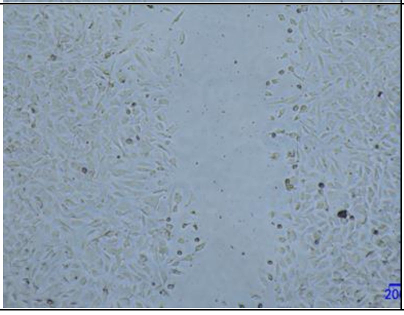	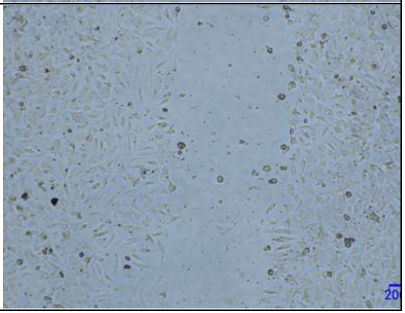

Figure S2. Six extracts (B1-B6) and five core components from CA inhibit the vascular tube formation of HUVECs compared to resveratrol as a positive control.

number	A1	A2	A3
Normal	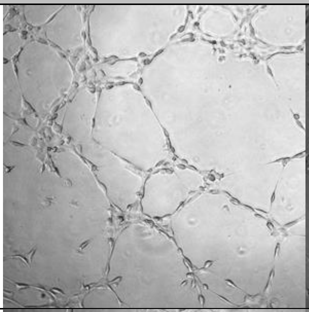	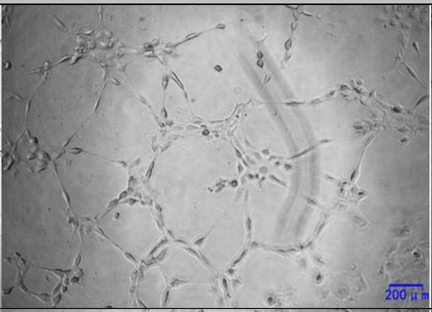	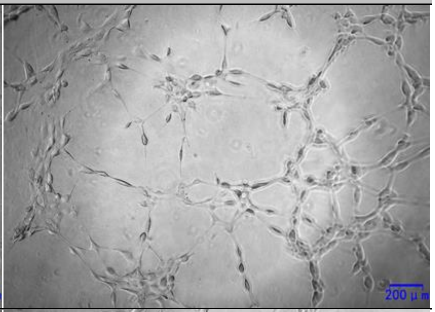
Model	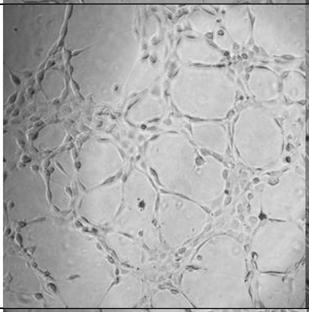	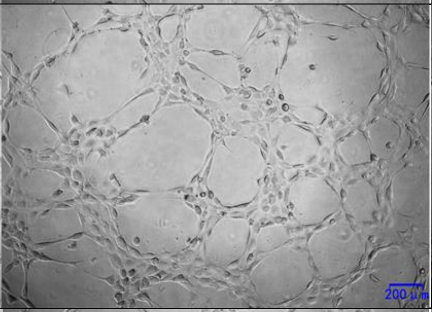	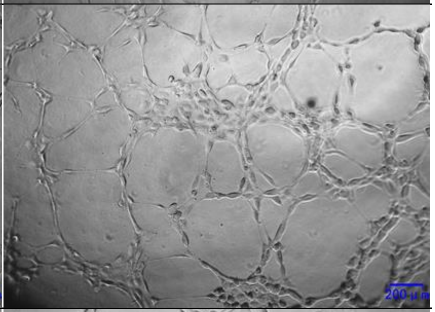
B1-Low	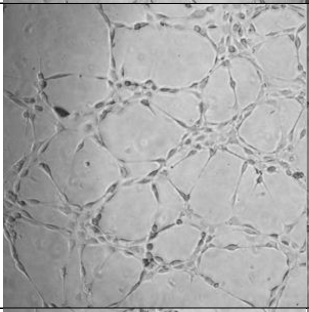	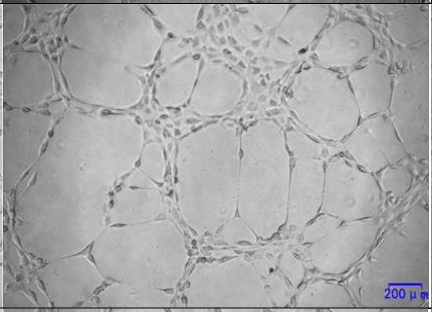	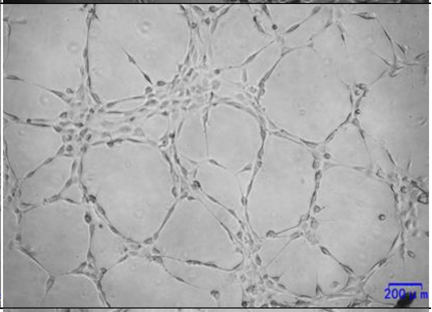
B1-Middle	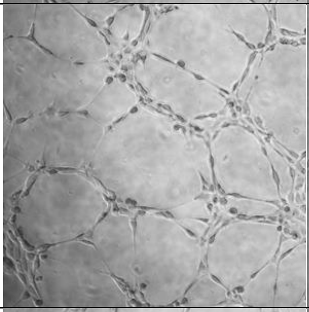	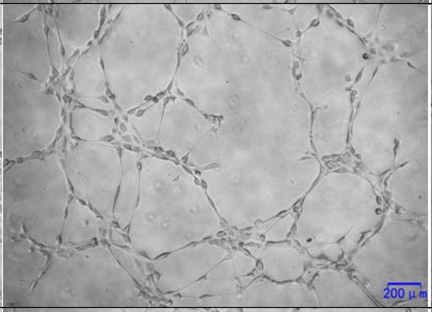	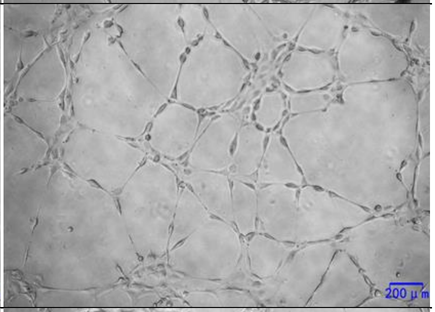
B1-High	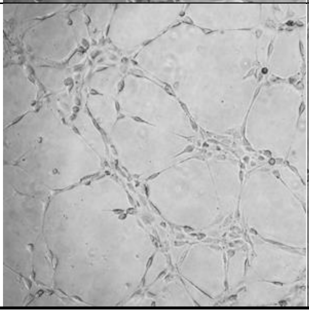	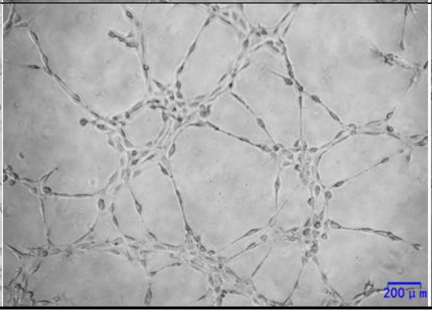	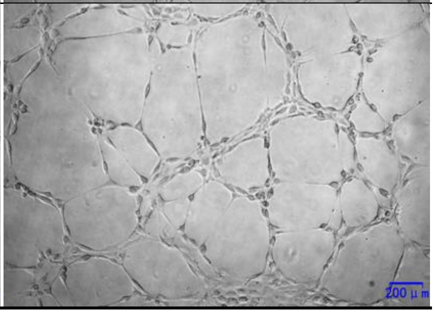

B2-Low	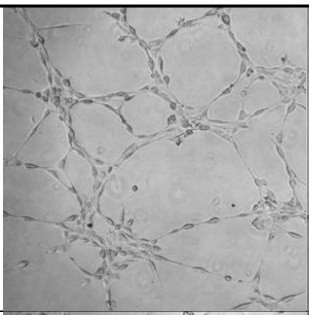	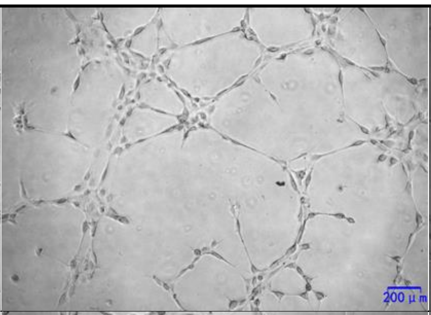	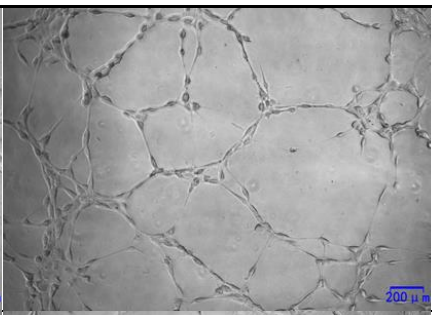
B2-Middle	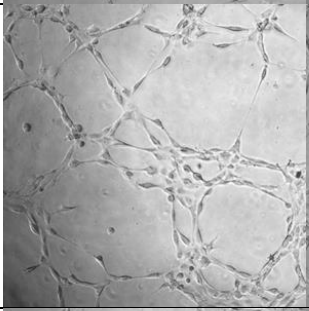	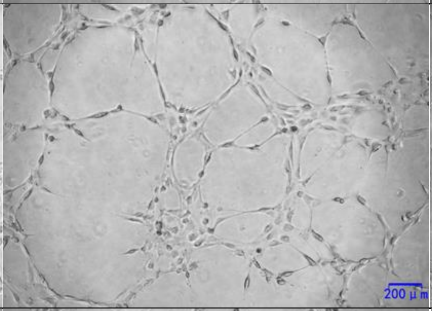	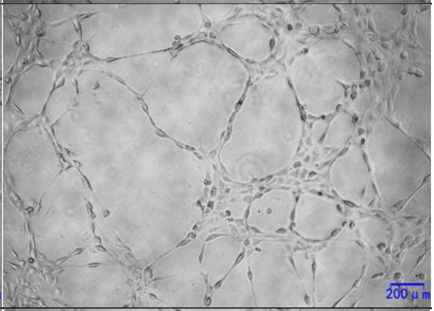
B2-High	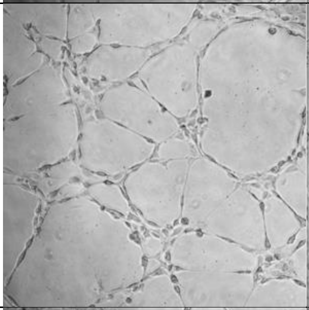	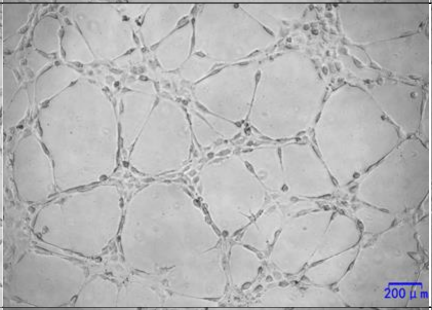	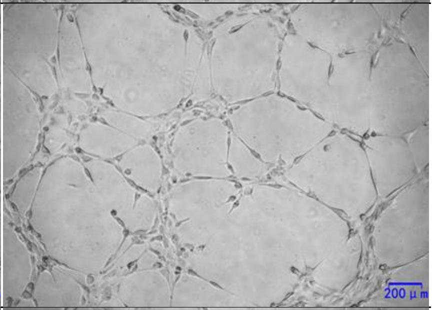
B3-Low	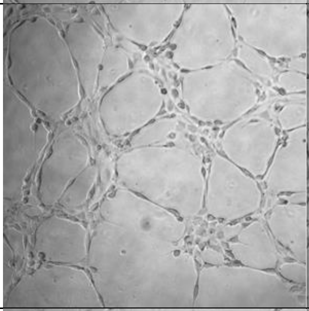	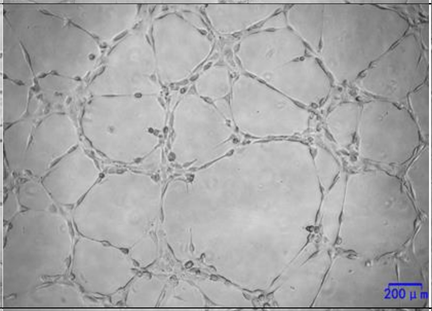	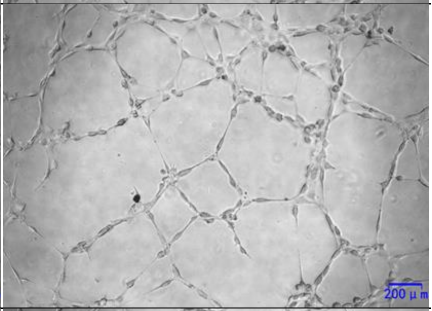
B3-Middle	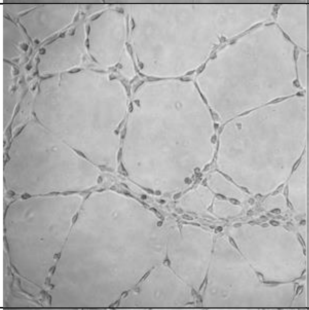	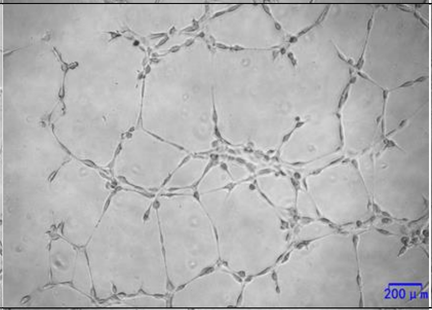	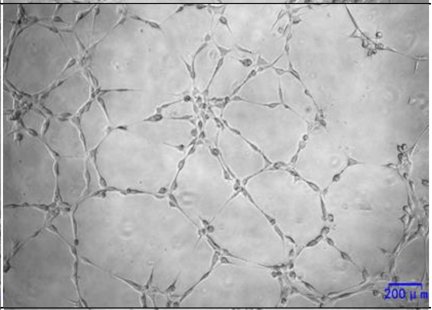
B3-High	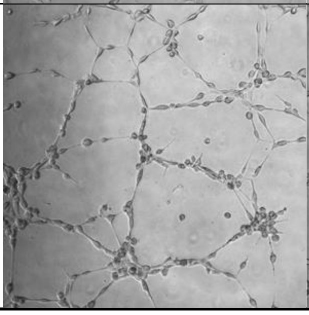	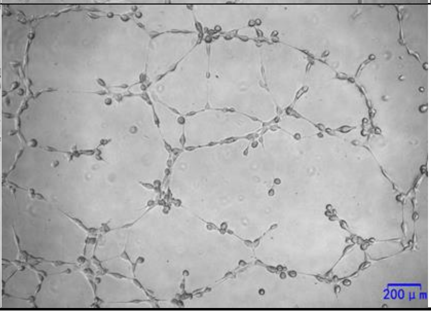	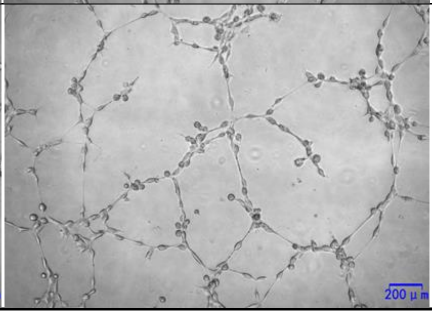

B4-Low	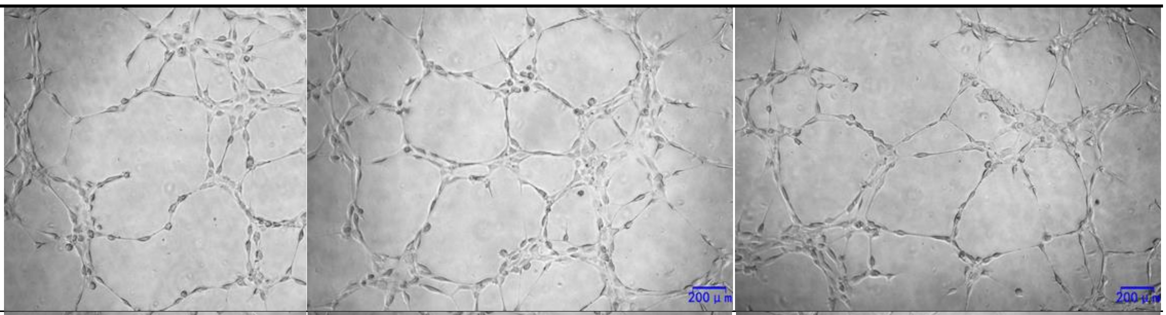
B4-Middle	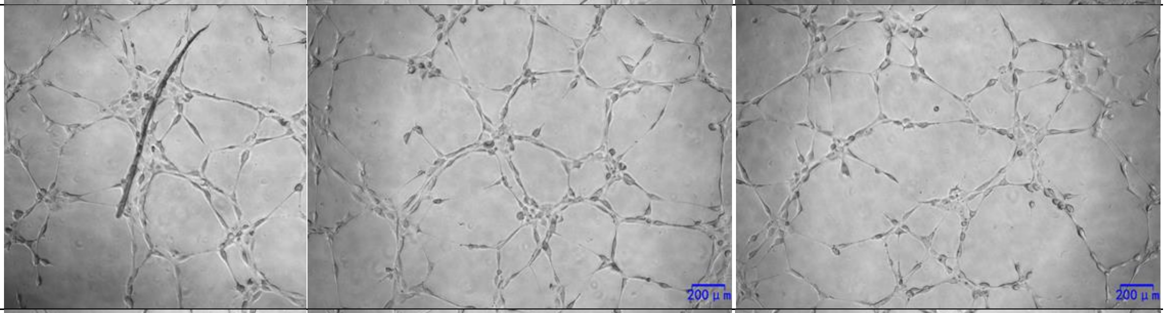
B4-High	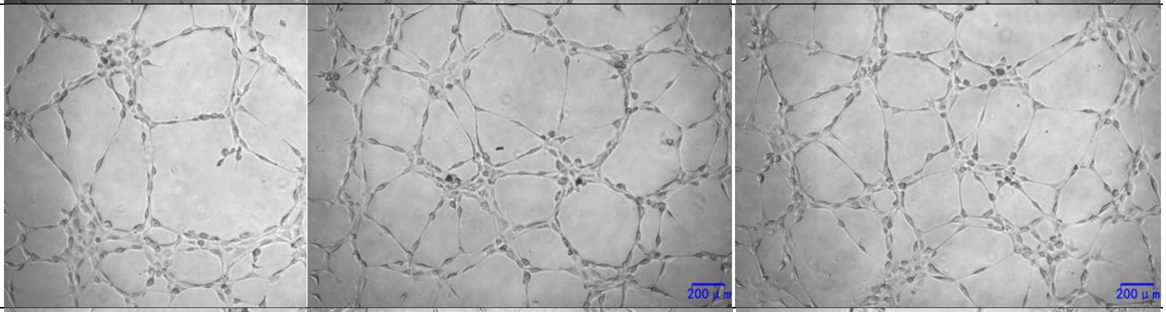
B5-Low	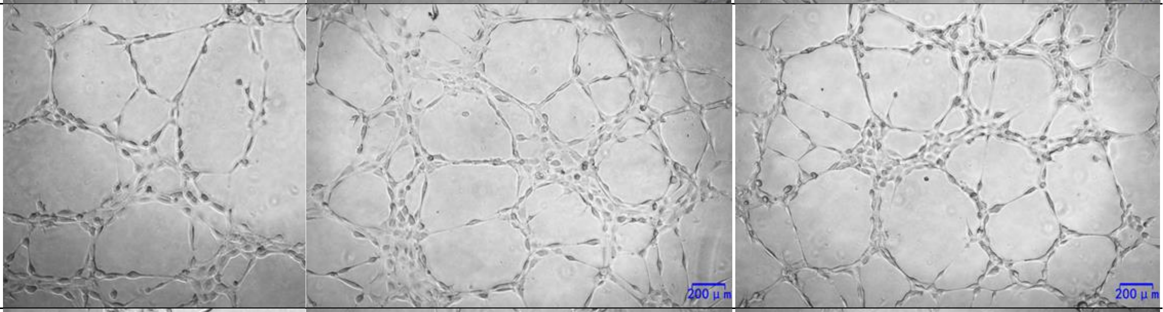
B5-Middle	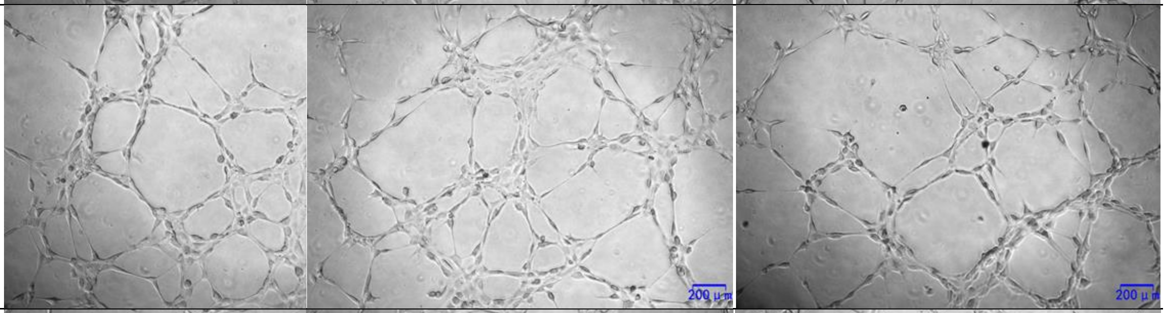
B5-High	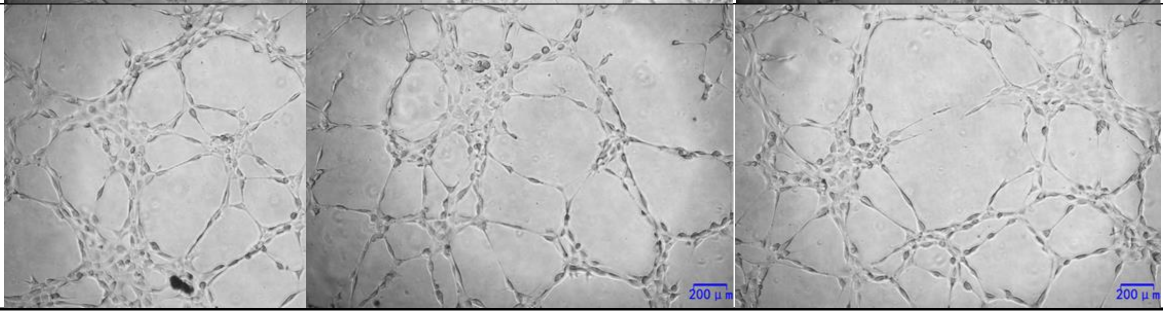

B6-Low	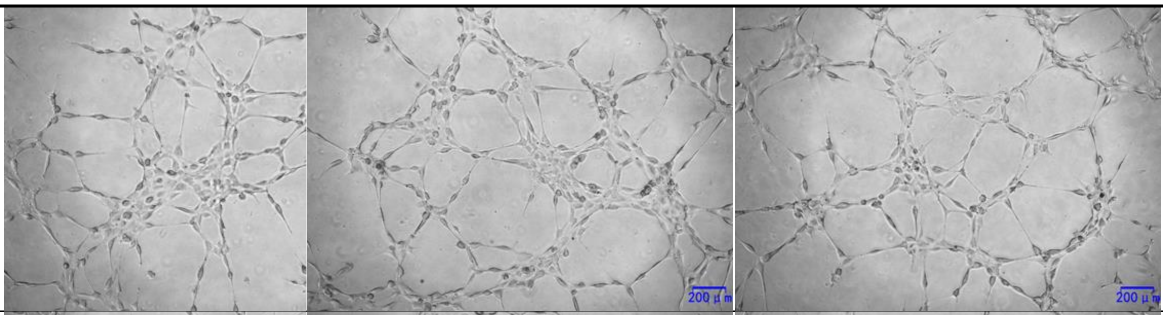
B6-Middle	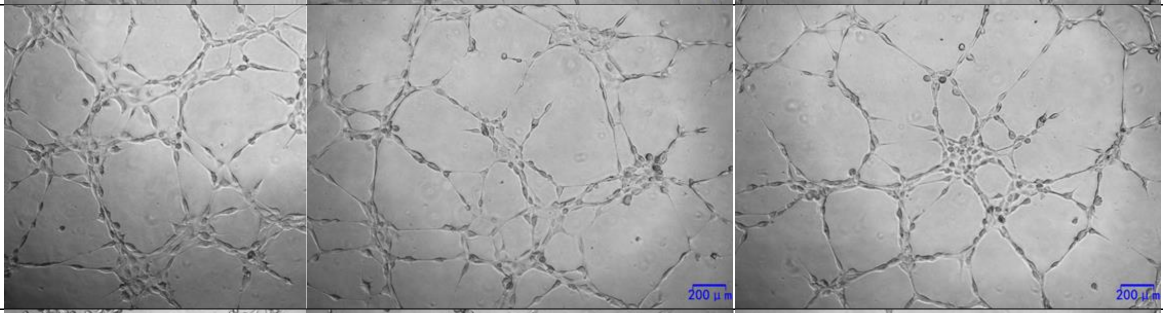
B6-High	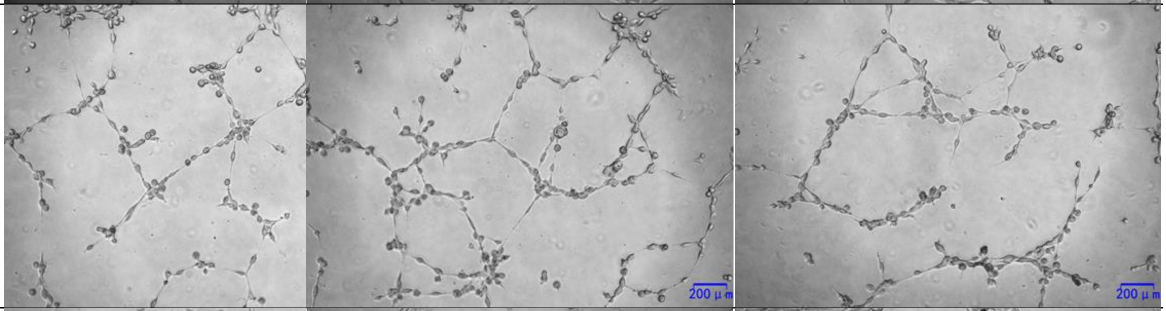
Asiaticoside-Low	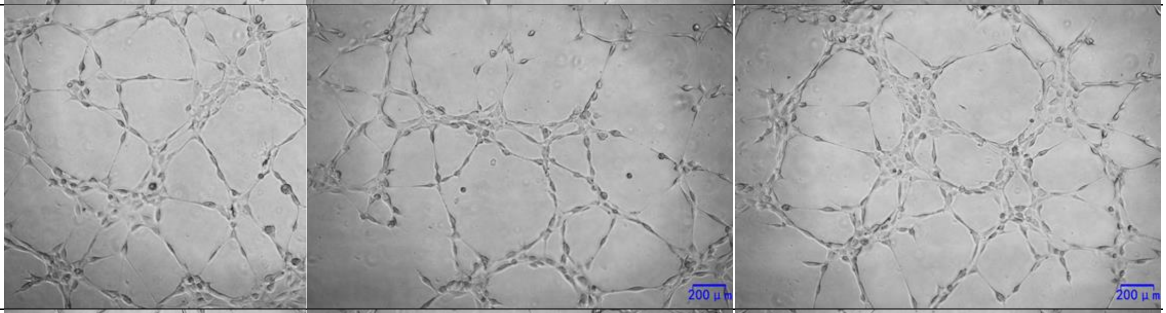
Asiaticoside-Middle	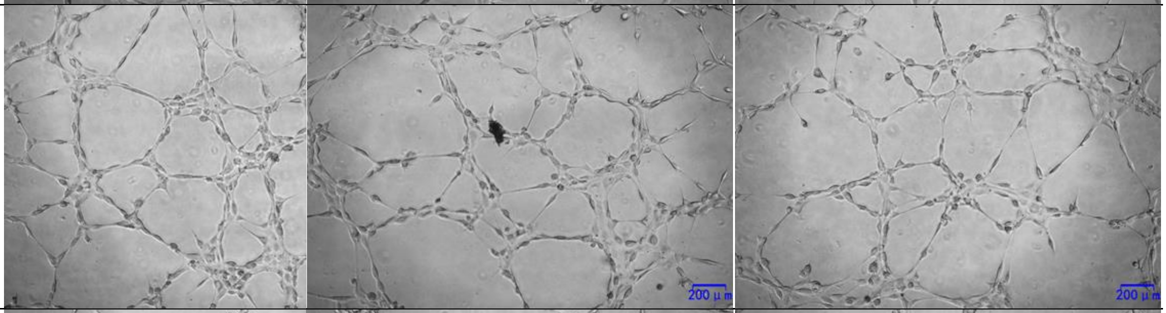
Asiaticoside-High	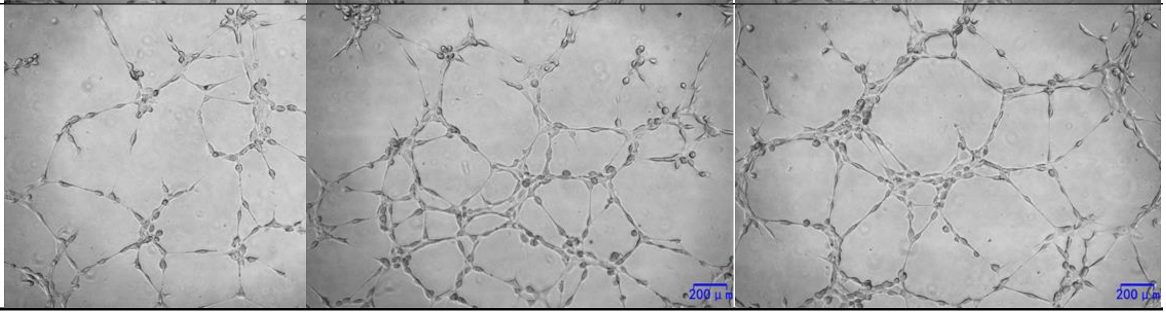

Asiaticoside B-Low	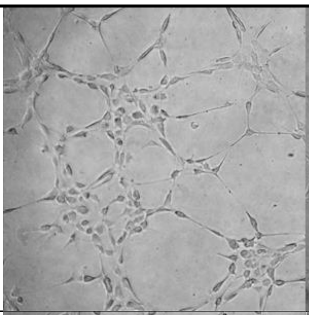	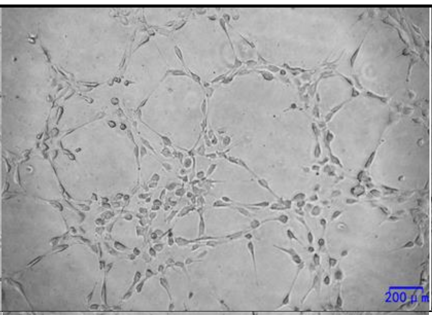	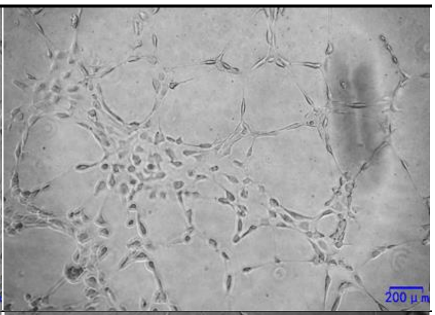
Asiaticoside B-Middle	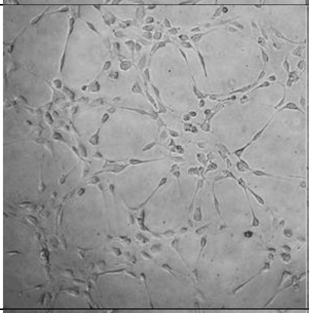	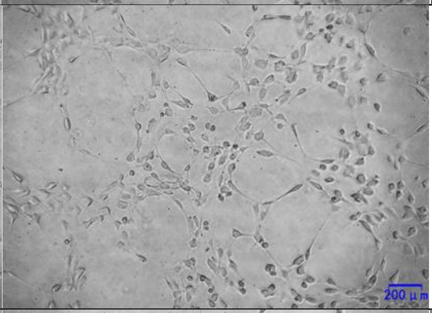	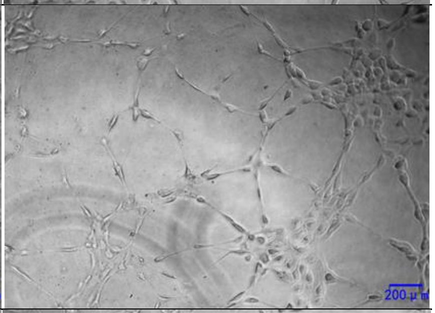
Asiaticoside B-High	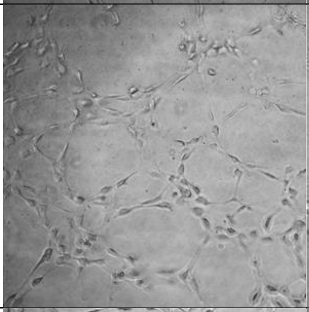	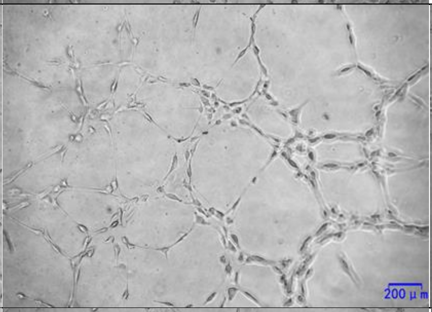	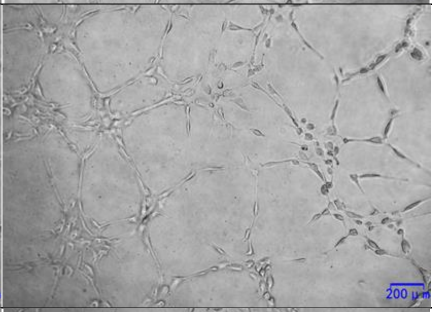
Asiatic acid-Low	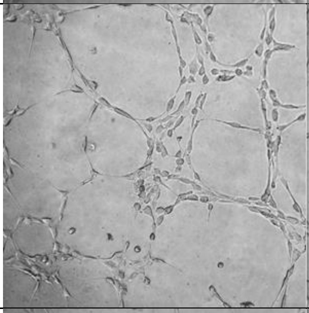	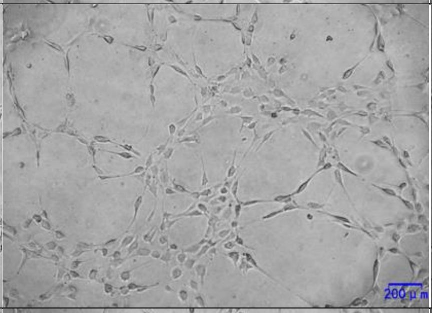	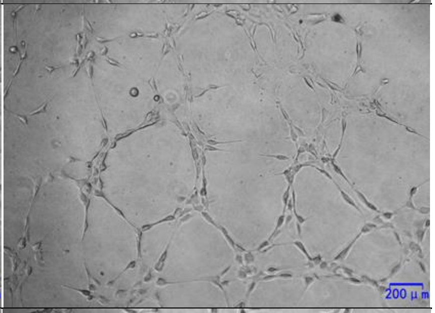
Asiatic acid-Middle	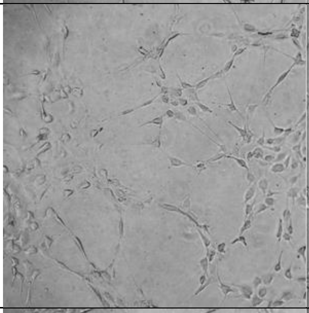	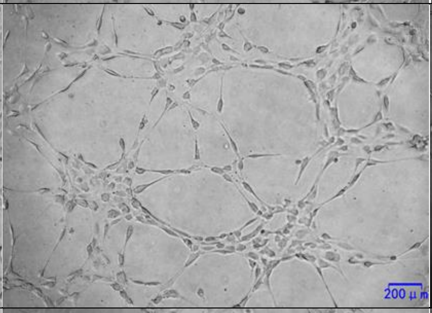	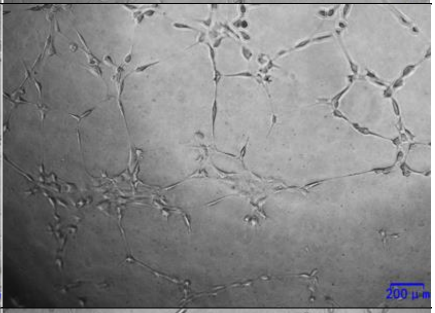
Asiatic acid-High	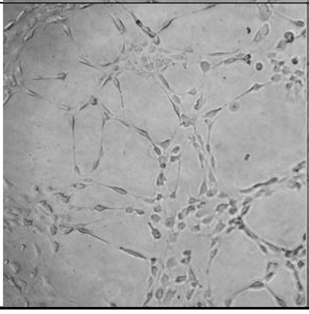	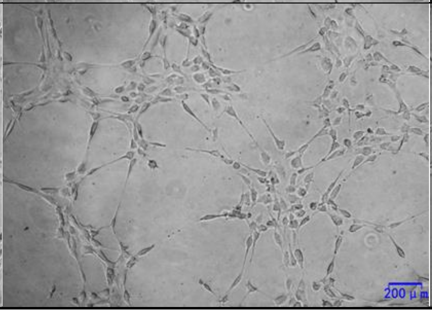	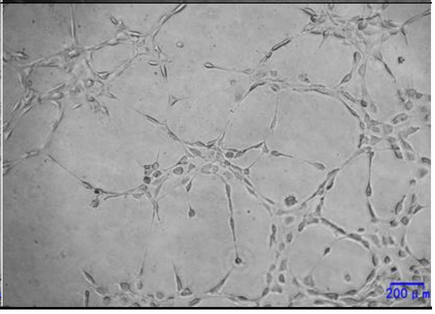

Madecassic acid - Low	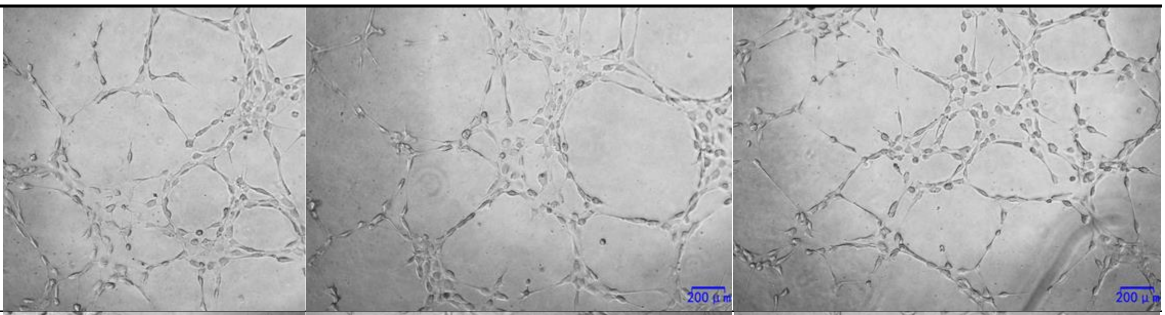
Madecassic acid - Middle	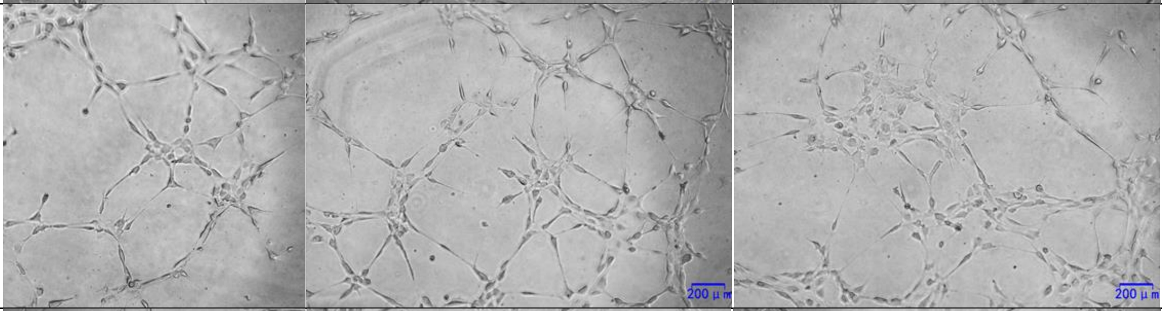
Madecassic acid - High	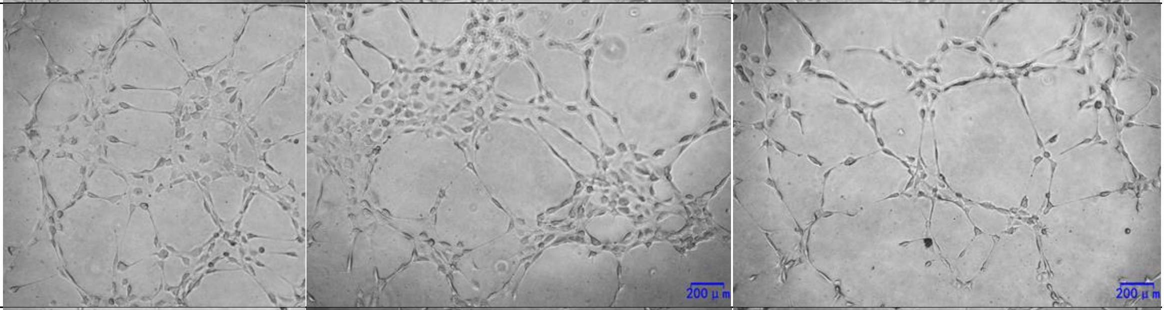
Madecassoside - Low	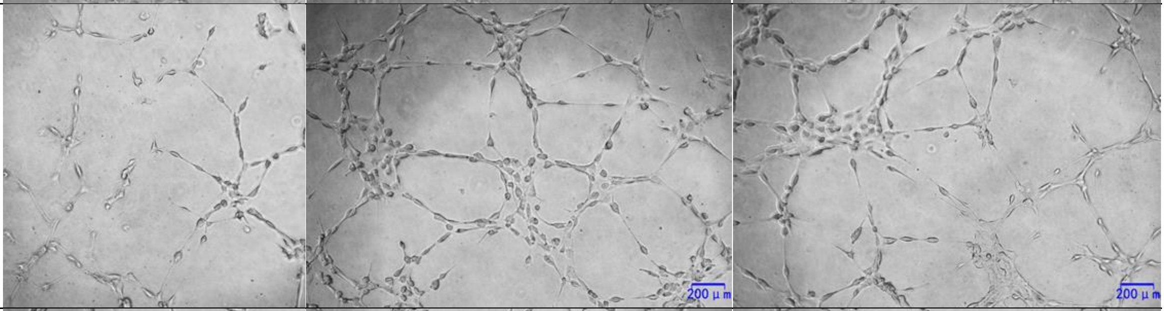
Madecassoside - Middle	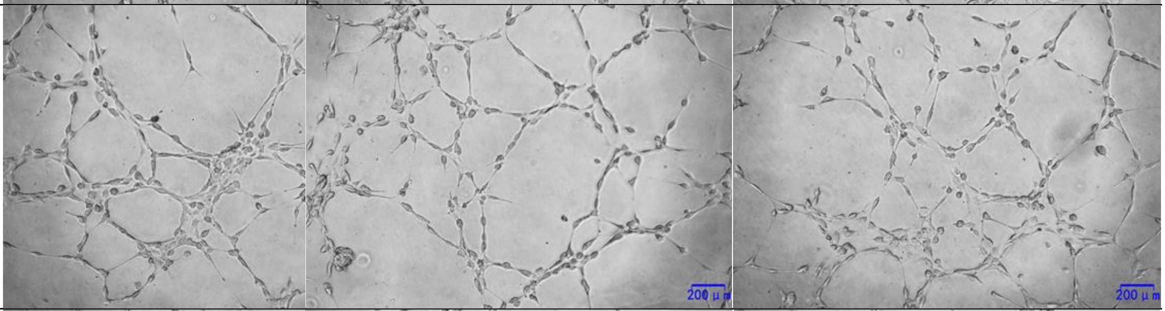
Madecassoside - High	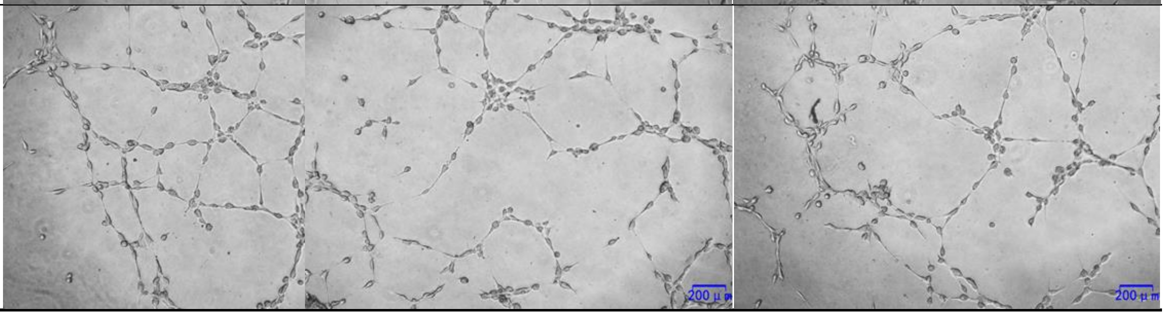

Rev-Low	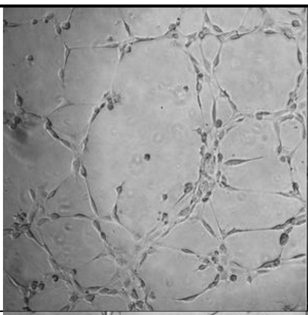	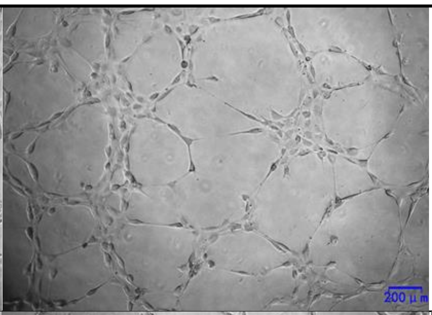	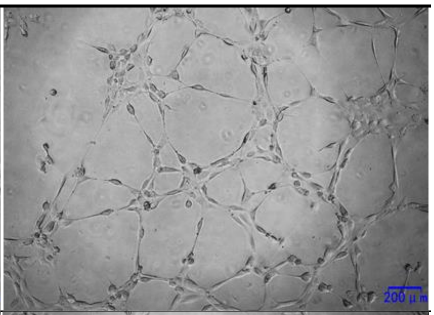
Rev-Middle	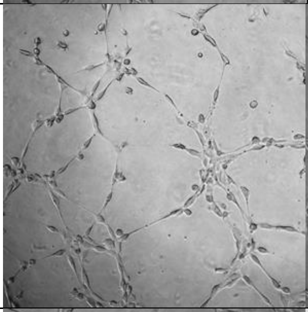	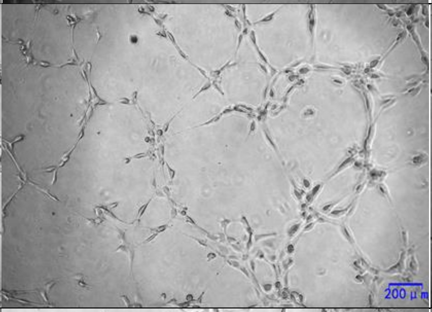	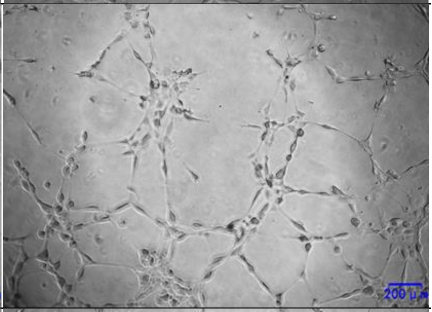
Rev-High	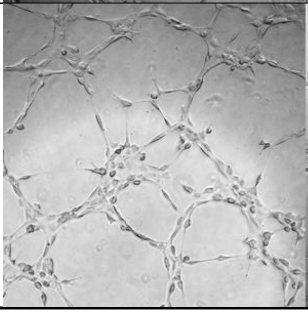	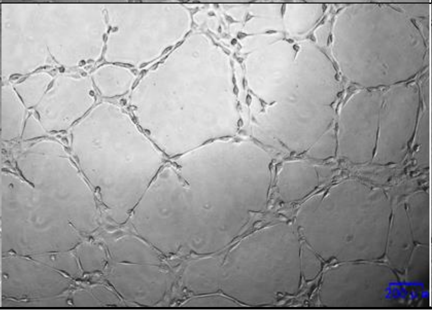	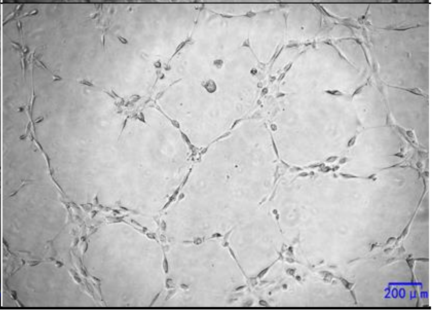
